# Nectar in oak savannas: implications for butterfly conservation

**DOI:** 10.1111/nph.70036

**Published:** 2025-03-09

**Authors:** Meigan Turner, Kevin E. McCluney, Ryan P. Walsh, Helen J. Michaels

**Affiliations:** ^1^ Department of Biological Sciences Bowling Green State University Bowling Green OH 43403 USA; ^2^ Department of Conservation The Toledo Zoological Society PO Box 140130 Toledo OH 43614‐0130 USA

**Keywords:** flower abundance, food‐resource estimate, habitat assessment, insect conservation, Karner Blue Butterfly, nectar quantity, Oak Savanna

## Abstract

- Restoring critically imperiled midwestern oak savanna habitat is frequently guided by the requirements of the federally endangered Karner blue butterfly (*Plebejus melissa samuelis*). Although studies often correlate butterfly population size or density with nectar species abundance, nectar characteristics are seldom considered.
- We compared floral nectar resources across 15 sites categorized by Karner blue butterfly occupancy by quantifying the floral abundance, nectar volume, and sugar concentration for 22 species, calculating their mean nectar availability per stem, measuring environmental variables, and estimating a species' nectar sugar contribution to the landscape across seasons.
- Species identity predicted nectar volume and sugar concentrations. Mean nectar volume ranged between 0.02 and 2.20 μl and sugar ranged between 3.06% and 61.26% Brix per flower. *Rubus flagellaris* and *Ceanothus americanus* contributed the most nectar sugar per stem in the spring and summer, respectively. Sites with a history of occupation had 11 times greater nectar sugar available in the spring than previous release locations or restorations without occupation.
- Improved understanding of seasonal distribution, quality, and abundance of oak savanna nectar resources is likely to aid habitat restoration planning and conservation efforts for pollinators of this critically imperiled habitat.

## Introduction

Oak savanna habitats historically covered at least 11 million acres of the Midwestern United States (Abella *et al*., 2020). Oak savanna habitats are characterized by sparsely dispersed mature oak trees with an understory of grasses and forbs, achieving a semi‐open canopy through fire and grazing disturbances (Olson, 1996; Sankaran *et al*., 2004; Anderson *et al*., 2007). Since European settlement, oak savannas have become highly fragmented due to fire suppression, agriculture, and urbanization (Nuzzo, 1986; Grossmann & Mladenoff, 2007) causing a severe decline in the diversity and abundance of native wildlife populations (Swengel & Swengel, 1999; Kocher & Williams, 2000; Meehan *et al*., 2013; Archer *et al*., 2014). With *c*. 0.02% of the historic range intact (Nuzzo, 1986) Midwest oak savannas are classified as a critically imperiled habitat in the United States (Noss *et al*., 1995). Oak savannas naturally preserve high levels of biodiversity relative to neighboring habitats (Leach & Givnish, 1999), making them an important focus for conservation efforts.

Habitat conservation can be guided by historical records (Landres *et al*., 1999; Swetnam *et al*., 1999), reference sites with minimal disturbance, or by the living requirements of an indicator species that requires high‐quality habitat to survive. A previously used indicator species for oak savanna habitats is the federally endangered Karner blue butterfly (*Plebejus melissa samuelis*) (U.S. Fish and Wildlife Service, 1992; Shuey, 1997; Chan & Packer, 2006). Due to the severe fragmentation of oak savannas and the intermediate flight ability of this small butterfly (King, 2003), conservationists have worked to improve the quality of remaining habitats and reintroduce populations. The success of Karner blue butterfly reintroduction depends on the suitability of local habitat, the characteristics of which are not yet fully understood (Pickens & Root, 2008; Walsh, 2017). Studies often relate butterfly abundance with host‐plant abundance (Fred & Brommer, 2003) or spatial distributions that limit search time (Crone & Schultz, 2022), nectar species abundance (Holl, 1995; Schultz & Dlugosch, 1999), and the area of the habitat (Moilanen & Hanski, 1998; Bergman & Kindvall, 2004).

Vegetation surveys evaluating the flowering plants available to pollinators commonly measure the density of flowering stems within a management unit (Williams, 1988; Chan & Packer, 2006; Walsh, 2017). Estimating resource availability by plant density, or even individual flower numbers, can lead to errors by not considering the nectar content each plant and/or flower can provide. For example, Schultz & Dlugosch (1999) determined that the abundance of Fender's blue butterfly (*Icaricia icarioides fender*) was not predicted by the abundance of individual flowers but by the total volume of nectar sugar from all native species present. Karner blue butterflies thrive in disturbed and semi‐open oak savannas that contain their exclusive larval host plant wild lupine (*Lupinus perennis*) (Opler & Malilul, 1998). First‐generation Karner blue larvae hatch in April and emerge as adult butterflies in early May and June. These adults will feed on nectar, mate, and lay eggs that produce adults in July and August (Grundel *et al*., 2000). While larval nutritional requirements are fairly well studied, adult nectar requirements of the Karner blue butterfly are less understood.

Nectar is mostly composed of water (35–85%; Seeley, 2009) and thus likely helps pollinators maintain hydration (Nicolson, 2009). However, it also contains nutritional compounds, such as sugars, lipids, and amino acids (Nicolson & Thornburg, 2007; Willmer, 2011; Cahenzli & Erhardt, 2012a). Different plant species can vary in the concentration and composition of nectar sugars (Baker & Baker, 1975; Dafni, 1992; Kearns & Inouye, 1993; Nicolson & Thornburg, 2007). Nectar is derived from photosynthesis, and therefore, nectar composition varies depending on a plant's exposure to light, water, and temperature (Freeman & Head, 1990; Pacini *et al*., 2003; Gallagher & Campbell, 2017). However, even with variations in nectar production due to environmental differences or plant age, relative uniformity of nectar composition within a species is expected (Nicolson & Thornburg, 2007).

The aim of this study is to provide a better understanding of how nectar resource distribution in space and time may influence habitat quality for the Karner blue butterfly and other nectar‐consuming pollinators within oak savanna habitats. Our approach was to couple nectar quality characteristics and floral abundance data for 22 oak savanna forbs with existing nectar plant density data in oak savanna habitats to examine whether sites categorized by Karner blue butterfly occupancy differ in nectar resource quality due to species‐specific variation in relative abundance and floral nectar traits. Specifically, we focused on the following questions. (1) How do environmental factors affect nectar composition, and can nectar quality be reliably associated with species identity? (2) Which species contributes the most sugar and hydration potential when considering variation in floral abundance, nectar volume, and sugar availability per stem? (3) How does the quality of habitat nectar resources (sugar density and total per site) vary among sites that differ in Karner blue occupancy or change between spring and summer seasons? To answer these questions, we quantified species nectar and floral characteristics, which were subsequently combined with previously determined flowering stem density estimates established by Walsh (2017) in oak savanna habitats associated with Karner blue butterfly conservation. Understanding these characteristics and seasonal variations in nectar resources will aid habitat restoration planning and benefit conservation efforts for nectar‐feeding pollinators of this critically imperiled habitat.

## Materials and Methods

### Nectar species selection

To select oak savanna species for nectar characterization, we utilized recent vegetation surveys of 15 oak savanna habitats conducted by Walsh (2017) within the Oak Openings Region of northwest Ohio (*n* = 8) and the Allegan State Game Area in western Michigan (*n* = 7). Because this vegetation survey was designed to better understand habitat characteristics conducive to Karner blue butterfly survival, sites had been categorized based on Karner blue presence: (1) currently occupied (OCC, in 2016)—before and after a drought in 2012, (2) formerly occupied (FOR)—no longer occupied after the drought, (3) previous release (REL)—previous release sites no longer occupied, and (4) priority restoration (RES)—high‐priority restoration sites for future releases (Supporting Information Table S2). All sites formerly occupied by the Karner blue were naturally occurring populations and not reintroduced by managers. Priority restoration sites were not associated with Karner blue occupancy but were assessed for oak savanna health and potential to become a future release location. We selected 22 flowering species (Table S1) from Walsh (2017) survey data for characterization of floral availability and nectar quality based upon a tiered set of criteria including frequency of species occurrence across sites (≥ 5 sites), comparison of stem density (≥ 2 stems m^−2^ quadrat), and if previous characterization of nectar quality was completed by Arnold & Michaels (2017). To evaluate whether species chosen for further study were representative of the standing nectar crop, we assessed the proportion of plants characterized for floral and nectar attributes within each site for each season (Table S3).

### Nectar sampling

Vegetation surveys at each site were conducted once in the spring and again in summer to assess patterns of potential nectar resource availability over time. Nectar volume and sugar concentration of each species were measured along with environmental variables to examine sources of variation in nectar quality within and among a subset of sites (Table S4). Because frequent rainfall events limited field measurements, nine species were raised in glasshouse conditions from locally collected native seed or transplants, while two species (*Ceanothus* americanus L. and *Rosa carolina* L.) were purchased to improve nectar collection sample sizes. To evaluate whether samples from glasshouse conditions are comparable to those from the field, nectar samples from the field and glasshouse were compared for two species (see Notes S1). Floral abundance and nectar data were collected between May and August in 2018 and 2019 from oak savanna sites in the Oak Openings Region of Toledo, Ohio, but this nectar information was applied to previous counts of stem density in the Allegan State Game Area, Michigan, due to time and funding constraints. Previous studies by Pamminger *et al*. (2019) on sunflower varieties concluded that microhabitat and geographic region have a significant but small influence on nectar quality. Nectar sampling locations in Ohio and Michigan, USA, are *c* 298 km apart, a distance that is also unlikely to influence nectar content, although site effects are expected.

Beginning at a random GPS coordinate within a property boundary, we placed a 25 m by 3 m wide transect through the nearest patch of selected flowering species, sampling plants from each transect only once and recording transect GPS coordinates. Sampled plants of the same species were a minimum of 0.5 m apart. Species too rare to appear in these transects were sampled when encountered. To prevent possible contamination or removal of floral resources by visitors (Wyatt *et al*., 1992), unopened or newly opened flowers were bagged with bridal‐mesh netting, which was removed for sampling within 24–48 h or when the flower had opened. If a rainfall event occurred, sampling was delayed 24 h to ensure that nectar was not washed out of the flower. Although flower age varied as a result, this approach sampled flowers that were typically 24–72 h old.

Environmental factors (temperature, relative humidity, soil moisture, and canopy cover) were recorded at the time of flower sampling to measure potential effects on nectar production. Local temperature and relative humidity were measured with a psychrometer (Mengshen Digital Thermometer & Hygrometer, M350). We used a time‐domain reflectometer (Spectrum Technologies Inc., Field Scout TDR 300) to determine the soil moisture at the base of each plant. Percent canopy cover, a proxy for direct sunlight, was measured by canopy photography above each plant with the mobile application HabitApp (Mobile application, HabitApp v. 1.1, Scrufster). Because the sampling of some species was compromised by frequent rain events during flowering, we grew some species in the glasshouse at Bowling Green State University (Day, 2020).

We collected nectar with microcapillary tubes from mature, open flowers. For species with many small flowers, a centrifuge technique was used to remove nectar without damaging the floral tissue (Arnold & Michaels, 2017). Each single inflorescence was collected and stored on ice (Bertsch, 1983) until the entire transect was collected and samples could be processed onsite using a microcentrifuge (HF120 NanoFuge with 6 × 1.5‐ml rotor; Tomy Seiko Co., Tokyo, Japan) powered by a car inverter (EverStart plus 400 W, 120 W cigarette plug). Each inflorescence was placed face down into a 1.5 ml Eppendorf tube partially filled with glass wool (0.15 g ± 0.05), leaving 5 mm of space in the bottom. To increase the probability of detecting trace amounts of nectar, the maximum number of flowers that could fit in a single layer was placed face down within the Eppendorf tube. Samples were spun at 6000 rpm (*c*. 2000 ***g***) for 2 min, which pulled nectar from the flowers to the bottom of the Eppendorf tube and collected pollen in the glass wool to prevent contamination of nectar composition (Nicolson & Thornburg, 2007). Centrifuge spinning duration was first tested to optimize this procedure (Day, 2020). The number of open flowers was counted before centrifuging to allow calculation of the volume of nectar produced per flower. The glass wool was removed, and the Eppendorf tube was placed into another cooler with ice for transport to the Bowling Green State University lab. Total nectar volume was measured with a 2 μl (±0.05‐μl accuracy) or 10 μl (±0.5‐μl accuracy) micropipette depending on estimated sample volume. Sugar concentrations were estimated using a handheld refractometer (Bellingham + Stanley, model 45–81, 0–50% Brix, low volume) calibrated to grams of sucrose per 100 grams of solution (Brix) (Bolten *et al*., 1979). Nectar samples exceeding 50% Brix were diluted by 50% using distilled water (Kearns & Inouye, 1993).

### Species floral availability

Floral availability was defined as the number of flowers per stem accessible to a pollinator at any given time of encounter. When a species was encountered in the field, the total number of open flowers per stem in the ground was recorded. For species that presented relatively few (1–30) open flowers at a time, the total number of flowers per stem was counted (*Comandra umbellata* L., *Dianthus armeria* L., *Fragaria virginiana* Duchesne, *Hypericum perforatum* L., *Lithospermum canescens* Michx., *Potentilla simplex* Michx., *Rosa carolina*, and *Rubus flagellaris*). For other species with greater floral abundance, complicated floral arrangements, and/or many florets in a capitulum, as in the Asteraceae, floral units were estimated on each stem following the approaches in Schultz & Dlugosch (1999), Baldock *et al*. (2015) and Tew *et al.* (2021) by counting the total number of branches, the number of inflorescences available on three branches, and/or the number of open flowers available on three inflorescences present on the stem to scale up the values to totals per stem. Data collected throughout the season was averaged (mean) to estimate the number of flowers likely available to visitors.

### Nectar quality analysis

To evaluate potential resources for nectar‐feeding pollinators within the spring and summer vegetation surveys, we determined nectar volume (μl) and sugar concentration (Brix) per flower for each species. Nectar sugar content was converted from Brix to mg of sugar following (Bolten *et al*., 1979; Hicks *et al*., 2016). For each species, the mean nectar volume (μl) and sugar (mg) per flower were multiplied by the mean number of flowers available per stem. Species‐specific nectar volume and sugar availability per stem were compared to determine potential sugar provisions and hydration potential of each species for effective pollinator conservation.

Species‐specific sugar per stem estimates were applied directly to the number of stems recorded within the Walsh (2017) spring and summer vegetation surveys to estimate quadrat‐level nectar resources. Plants not characterized for nectar quality, not identified to species, or recorded as ‘Unknown’ were not included in determining mean quadrat nectar content. All non‐flowering quadrats were recorded as providing zero resources. The total number of quadrats surveyed within a site was used as a proxy measurement for site size because one transect was randomly placed for every 850 m^2^ of habitat. Examining resources per quadrat allowed us to evaluate resources at the scale nectar feeders would encounter them in the field.

We applied mean stem level resources for each species to the number of stems present in the entire survey area of each site and estimated site total nectar quality by summing the nectar volume and sugar contribution of all species. Site total nectar volume (TV_a_) and sugar availability (TS_a_) were estimated during each season where TV_a_ = Σ_*i*_ = 1(*V*_*i*_ × *N*_*i*_) and TS_a_ = Σ_*i*_ = 1(*S*_*i*_ × *N*_*i*_), where *V*_*i*_ is mean volume and *S*_*i*_ is sugar available per stem for species *i*, and *N*_*i*_ is the number of stems within site *a* corresponding to species *i*. The total number of quadrats surveyed was incorporated into modeling to compare total resources available among sites. Similar upscaling of flower‐level nectar quality measures to the landscape level has been conducted by Schultz & Dlugosch (1999), Hicks *et al*. (2016), and Pamminger *et al*. (2019).

### Statistical analysis

Unless otherwise noted, all analyses were performed using JMP (JMP®, Student Edition 14, SAS Institute Inc., Cary, NC, 2019). Residual plots and normal quantile plots were used to assess assumptions of normally distributed errors and equal variance, and Log_10_ transformations were used to improve congruence with assumptions. These transformations were effective for all models. Analysis of environmental effects on nectar composition of Ohio field samples was completed for 13 of 22 species (excluding seven species previously characterized by Arnold & Michaels (2017) that lacked corresponding environmental data). To assess multicollinearity, we used non‐parametric Spearman's correlation to explore relationships between environmental variables and nectar metrics. A generalized linear model was used to evaluate the relationship between environmental factors (canopy cover, relative humidity, temperature and soil moisture) and dependent variables (nectar volume and sugar). To determine which environmental factors were most related to nectar volume and sugar concentration, we created a set of potential models with various combinations of predictors and compared them using AICc (corrected Akaike's Information Criterion). For each response, we identified the model with the lowest AICc value with a difference greater than two units from others (Burnham & Anderson, 2002). To evaluate how nectar volume (μl) and sugar availability (mg) per plant may be affected by environmental factors, we also used combinations of environmental predictors to compare models predicting species‐specific nectar quality per stem based on the nectar volume (μl) and sugar concentration (Brix) per flower and their estimated floral abundance. We additionally compared the quality of nectar samples between our study and those in Arnold & Michaels (2017) as well as sampling locations (field vs glasshouse) to confirm consistent sampling methods (see Notes S1).

Flowering community composition was compared between site categories (OCC, FOR, REL, and RES) through non‐metric multidimensional scaling (NMDS) using the ‘metaMDS’ function in the vegan package from R v.3.4.3, using Bray–Curtis distances (Oksanen *et al*., 2018). The function in ‘vegan’ transforms the data if necessary and standardizes the scaling of the results to improve interpretation. Ordination plots for spring and summer vegetation surveys were based on the mean density of all flowering species recorded. We ran permutational multivariate analysis of variance (PERMANOVA) using the vegan package from R v.3.4.3, using Bray–Curtis distances (Oksanen *et al*., 2018) to test for significant differences in community composition between site categories within each season.

All 22 characterized species were included to assess quadrat and site‐level resources. To assess the effects of site category and season on the availability of sugar (mg) per 0.5 m^2^ quadrat and on the total sugar availability within site levels, we used linear mixed models with site as a random block effect. The best models were determined by p‐value, with the significance of the model and each fixed effect established through Kenward–Roger approximation (Bolker *et al*., 2009). We excluded any quadrats for which < 90% of flowering stems had been characterized to minimize potential measurement error. Category and seasonal availability of flowering stems and sugar per quadrat were compared between site categories and seasons using Tukey HSD all‐pairs comparisons.

## Results

### Nectar variation

Across all nectar samples, volume and sugar concentration per flower were not significantly correlated (Table S5, Spearman's *r*s = 0.009, *P* = 0.87). When species‐specific nectar volume (μl) and sugar availability (mg) per stem were applied directly to the number of stems recorded within vegetation surveys, a strong correlation was observed between total nectar volume and sugar availability across sites (Pearson's *r* = 0.98, *P* < 0.0001). This means that total sugar availability was highly correlated with total nectar volume and the two measures of nectar resources cannot be disentangled. Corresponding nectar volume analyses are reported within the modeling tables.

Nectar volume and sugar concentration per flower were best predicted by species identity, relative humidity, and canopy cover (nectar volume *F*_14,77_ = 23.75, *P* < 0.0001, Adj *R* ^2^ = 0.78, AICc = 128.47, Table S6a; sugar per flower: *F*_14,75_ = 8.03, *P* < 0.0001, Adj *R* ^2^ = 0.52, AICc = 93.75, Table S6b). The inclusion of sampling site as a block did not improve either model's AICc value, suggesting site‐specific environmental effects not recorded were a negligible factor influencing nectar quality per flower. Species identity was an important predictor of both nectar volume and sugar concentration per flower (*P* < 0.0001, LogW = 21.59, Table S6a; *P* < 0.0001, LogW = 7.57, Table S6b). As expected, an increase in relative humidity was associated with increased nectar volume and slightly decreased sugar concentration, likely due to reduced nectar evaporation (LogW = 4.74, *P* < 0.0001, Table S6a, LogW = 2.02, *P* = 0.009, Table S6b). Sugar concentration decreased weakly as canopy cover increased (*P* = 0.0008, Table S6b).

When nectar quality per flower was incorporated with floral display size (species mean number of open flowers per stem) to analyze predictors of nectar volume (μl) and sugar availability (mg) on a per stem basis, we found similar results (Volume: *F*_14,77_ = 8.98, *P* < 0.0001, Adj *R* ^2^ = 0.55, AICc = 128.47, Table S7a; Sugar: *F*_14,75_ = 18.09, *P* < 0.0001, Adj *R* ^2^ = 0.72, AICc = 146.58, Table S7b). Linear regression models confirmed that species identity was an important predictor of nectar volume and sugar availability, beyond environmental factors (*P* < 0.0001, LogW = 8.17, Table S7a; *P* < 0.0001, LogW = 18.19, Table S7b). Nectar volume per stem remained significantly influenced by relative humidity (*P* < 0.0001, LogW = 4.74) but not by canopy cover (*P* = 0.15, Table S7a). Sugar per stem was significantly decreased by canopy cover (*P* = 0.05, Table S7b). Overall, species identity had a large influence in predicting variation in nectar volume and sugar availability per stem.

### Species characterization

**Fig. 1:**
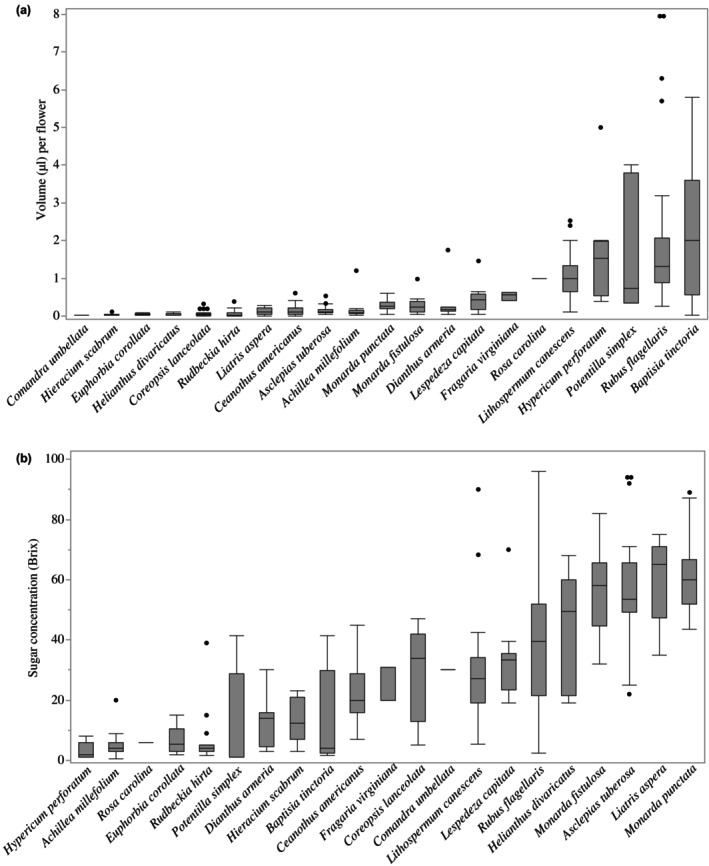
(a) Variation in total volume (μl) and (b) sugar concentration (Brix = g sucrose/100 g solution) per flower among species, ordered from lowest median to greatest. A mean of 15 samples were collected per species but ranging from 1 to 52 nectar samples. Box plot outlines display the first quartile and third quartile with the median value as a horizontal line within. Whiskers above and below represent the minimum and maximum values, excluding outliers, of nectar sample variation within that species, while dots indicate outliers. Sample sizes were < 5 for three species (*Comandra umbellata*, *Fragaria virginiana*, *Rosa carolina*).

We collected a mean of 15 nectar samples per species, with a mean nectar volume ranging from 0.02 to 2.20 μl per flower (Table S8; Fig. 1a). *Baptisia tinctoria* and *Rubus flagellaris* had the greatest nectar volume per flower (2.20 and 1.83 μl, respectively). The largest volumes were *c*. 100 times greater than the smallest nectar volumes of *Comandra umbellata* and *Dianthus armeria* (0.02 and 0.38 μl, respectively). Species mean sugar concentration ranged from 3.06–61.26% Brix (Fig. 1b). *Monarda punctata a*nd *Liatris aspera* had the highest sugar concentrations (61.26 and 59.75% Brix, respectively). These highest sugar concentrations were *c*. 15 times greater than those of *Hypericum perforatum* and *Achillea millefolium* (3.06 and 5.33% Brix, respectively). Nectar removal was consistently unsuccessful for *Krigia virginica* via microcapillary tubes, centrifugation, or paper wicks.

### Species contribution to nectar availability

When species‐specific nectar volume (μl) and sugar availability (mg) per stem were applied directly to the number of stems recorded within vegetation surveys, a strong correlation was observed between total nectar volume and sugar availability across sites (Pearson's *r* = 0.98, *P* < 0.0001). This means that total sugar availability was highly correlated with total nectar volume and the two measures of nectar resources cannot be disentangled. Corresponding nectar volume analyses are reported within the modeling tables.

**Fig. 2:**
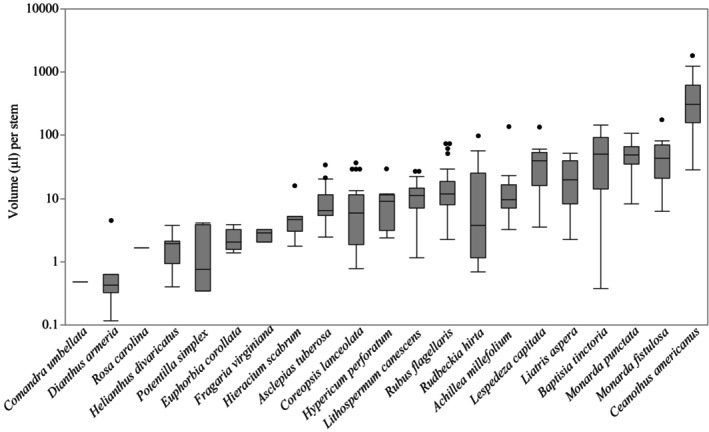
Variation in total volume (μl) per stem among species, ordered from lowest median volume to greatest. Box plot outlines display the first and third quartiles with the median value as a horizontal line within. Whiskers above and below represent the minimum and maximum values of nectar sample volume, excluding outliers, while dots represent outliers.

**Fig. 3:**
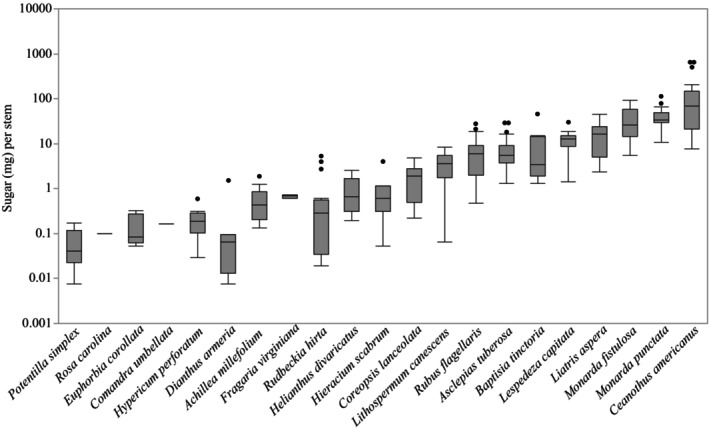
Variation in total sugar (mg) per stem among species, ordered from lowest median sugar availability to greatest. Box plot outlines display the first and third quartiles with the median value as a horizontal line within. Whiskers above and below represent the minimum and maximum of total sugar per stem within that species, excluding outliers, while dots represent outliers.

Estimates of nectar volume and sugar concentration per stem in the ground for each species provided insights into nectar resources that will be particularly valuable for habitat restoration. For example, *Ceanothus americanus* is a shrub that provided the most flowers per stem (Table S9). In the field, full‐size plants were a mean of 2–3 feet high. Each inflorescence held *c*. 120 flowers, producing an estimated 3001 flowers per stem in the ground. With this extraordinarily high floral availability and moderate sugar per flower (Fig. 1b), *C. americanus* provided the most nectar volume and sugar per stem (442.79 μl, Fig. 2; 134.20 mg, Fig. 3). The species providing the second‐greatest number of flowers was *Rudbeckia hirta*, with an estimated 255 flowers per stem, while the lowest was *Potentilla simplex*, which displayed a mean of one flower. Also noteworthy are *Baptisia tinctoria* (55.75 μl), the species with the second‐greatest nectar volume per stem, and *Monarda punctata*, which provided the next‐highest sugar content per stem (40.84 mg). By contrast, at the opposite end of the range, eight of the 22 species provided < 3 μl of nectar and < 1 mg of sugar per stem in the ground (*Comandra umbellata*, *Dianthus armeria*, *Euphorbia corollata*, *Fragaria virginiana*, *Helianthus divaricatus*, *Krigia virginica*, *Potentilla simplex*, and *Rosa carolina*).

### Site assessment

Comparisons of flowering community composition for spring and summer (based on the mean density of all flowering species, *n* = 36 and *n* = 45, respectively) between site categories revealed interesting patterns. Spring flowering community composition was significantly different between category types (df = 14, *F* = 1.88, *P* = 0.03, Table S10a; Fig. S1). Sites OCC and FOR by the Karner blue appeared to have more similar spring flowering communities than REL locations and RES sites. Interestingly, the OCC site closest in the ordination to REL and RES sites was the only active Karner blue butterfly habitat in the Oak Openings region of Ohio at the time of this study and was extirpated by 2019 (Walsh, pers. com.). For summer, we found a non‐significant trend for OCC and FOR sites to be oriented away from REL and RES sites in ordination space (df = 14, *F* = 1.37, *P* = 0.09, Table S10b; Fig. S2).

Walsh (2017) found no significant difference in nectar plant density between sites OCC and FOR by the Karner blue, but that both had greater nectar plant densities than REL sites. When we analyzed our 2018–2019 survey data from each season independently, there was no difference in the number of flowering stems per 0.5 m^2^ quadrat among categories in the spring (Wilcoxon/Kruskal–Wallis test, *P* = 0.30) but we found a significant difference in the summer (*P* < 0.0001, Table S11). Further analysis of summer floral stem density revealed RES sites had significantly more flowering stems (Tukey–Kramer HSD all pairs comparison, Table S12) and fewer empty, non‐flowering quadrats than other categories (22%, Table S13). Examination of changes in flowering stem abundance between seasons within a category type (Table S14) showed that currently OCC and REL sites had significantly greater flowering stem density in the spring than summer (Wilcoxon each‐pairs comparison, *P* < 0.0001 and *P* = 0.003, respectively). No difference was found in flowering stem density between seasons of FOR sites (*P* = 0.36) or RES sites (*P* = 0.51).

**Fig. 4:**
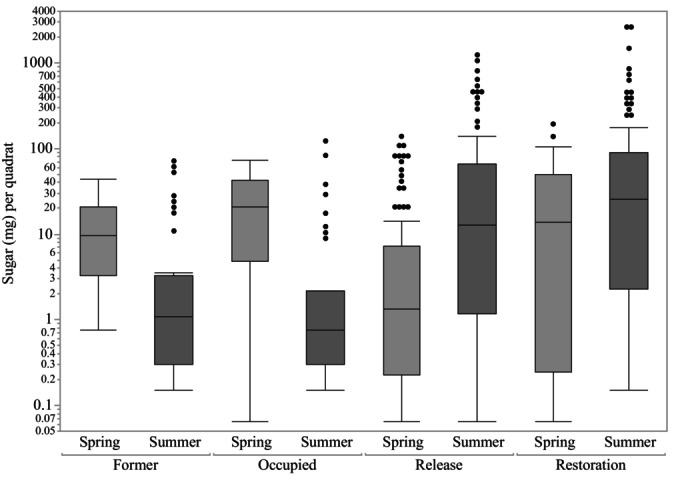
Seasonal median sugar (mg) per 0.5 m^2^ quadrat within sites categorized as former (FOR), occupied (OCC), previous release (REL), and priority restoration (RES). In both seasons, OCC and FOR sites are statistically similar (Table S17) as were REL and RES sites. Box plot outlines display the first and third quartiles with the median value as a horizontal line within. Whiskers above and below represent the maximum and mnimum, excluding outliers, total sugar per quadrat within a category/season, while dots represent outliers.

Comparisons of the number of flowering stems and nectar sugar (mg) availability per quadrat within site categories across both seasons found that OCC sites by the Karner blue butterfly had the strongest correlation between flowering stems and sugar per quadrat (Spearman's correlation rs = 0.75, *P* < 0.0001), while REL sites had the weakest (*r*s = 0.48, *P* < 0.0001). Total nectar sugar availability (mg) per 0.5 m^2^ differed by site category and season (*F* = 33.72, *P* < 0.0001, Adj *R* ^2^ = 0.16, AICc = 1682.57, Table S15a). Display of sugar dynamics across seasons (Fig. 4) suggests that sites with a history of occupation (FOR and OCC) have more sugar in spring and a decline in summer (median sugar (mg per quadrat) in FOR: spring 9.68 vs summer 1.09 mg per quadrat; OCC: 20.71 spring vs summer 0.76), while most REL and RES sites showed the opposite pattern (median sugar (mg per quadrat) in REL sites: spring 0.68 vs summer 12.21; RES: 4.91 in spring vs summer 25.44). However, as expected from environmental factors driving photosynthesis, spring samples at all sites had less sugar per quadrat available than in the summer (spring mean = 20.55 mg (±1.9), summer mean = 92.42 mg (±18.23); *P* = 0.03). Site size did not have a significant influence on sugar availability per quadrat (*P* = 0.21). Similar to quadrat‐level analyses, total nectar sugar (g) within a site was best predicted by the interaction between category and season (Model *F*_7,22_ = 3.08, *P* = 0.005, Adj *R* ^2^ = 0.33, AICc = 83.48, Table S16).

Model parameter estimates showed that OCC contained the greatest spring total sugar available across categories. *Post hoc* testing to further analyze how resources vary among occupancy categories within each season (Tukey–Kramer HSD all pairs testing, Table S17) showed that spring samples from REL sites produced fewer flowering stems and lower sugar density (numbers or mg per 0.5 m^2^ quadrat) than OCC and FOR sites. In the spring, sites with a history of Karner blue butterfly occupation (OCC and FOR) provided a median sugar density 11 times greater than REL or RES sites (Fig. 4). When considering species that occurred in the spring vegetation survey, *Rubus flagellaris* provided the highest sugar concentration and the most nectar sugar per stem (Table S9; Fig. S3).

In the summer sampled communities, the magnitude of the difference between site nectar resources depended on the resource being measured. Among four pairwise category comparisons (OCC/REL, OCC/RES, FOR/REL, and FOR/RES) there was no difference in summer flowering stem density, but we found a significant difference among site categories when nectar sugar available per quadrat was analyzed (Table S17). Occupied and FOR sites had lower sugar availability in the summer compared to REL sites (*P* < 0.0001 and *P* < 0.0001, respectively) and RES sites (*P* < 0.0001 and *P* < 0.0001, respectively). These results indicate that species‐specific floral abundance and nectar sugar quality provided an improved assessment of the resources available and revealed associations not previously measured.

## Discussion

### Nectar variation: how do environmental factors affect nectar composition, and can nectar quality be reliably associated with species identity?

In both field and laboratory conditions, nectar sugar concentrations decline as temperature increases in some species (e.g. *Ipomopsis longiflora* Freeman & Head, 1990; Villarreal & Freeman, 1990). However, water stress generally results in reduced nectar volume and fewer and/or smaller flowers produced (Kuppler & Kotowska, 2021). Humidity conditions can also account for influences on nectar secretory changes due to volume equilibration with air moisture (Willmer, 2011). Nectar chemistry and production characteristics likely have strong genetic components (Kearns & Inouye, 1993). As plants age, the changes observed in sugar proportions tend to be minor compared to distinct species differences observed (Nicolson & van Wyk, 1998). Our findings are consistent with literature suggesting that species identity can reliably predict nectar volume and sugar concentrations, and environmental factors do not hide the consistent differences between species (Kearns & Inouye, 1993; Mitchell, 2004). Over the range of environmental conditions sampled, species identity was important and easily visible, despite the effects of environmental variation. Others have also found that relative humidity has a smaller effect than species identity (Farkas *et al*., 2012). The volume of nectar present within a flower decreased as local relative humidity decreased, likely because nectar evaporated to equilibrate with the surrounding air moisture (Bertsch, 1983; Wyatt *et al*., 1992; Willmer, 2011). Increased canopy slightly decreased floral sugar concentration, likely due to a decrease in photosynthesis and therefore sugar for nectar production (Pacini *et al*., 2003). Demonstrating species‐specific nectar traits further supports the application of nectar quality data to vegetation surveys within our study and its use in future habitat assessments. This allows prediction of nectar resources within a habitat from the identity of flowering species and stem density.

Little investigation has been completed for the heritability of other nectar traits such as concentration of sugars, amino acids, age effects, temporal patterns, taste, or scent (Mitchell, 2004; Parachnowitsch *et al*., 2019). Nectar production is a complex process, and our study just breaks the surface. Species‐specific nectar production can also be influenced by floral age (Kato & Sakai, 2008; Farkas *et al*., 2012), can vary daily, and has different 24‐h production patterns (Mohr & Jay, 1990; Pham‐Delègue *et al*., 1990; Burquez & Corbet, 1991; Gilbert *et al*., 1991; Stern & Gazit, 1996). Species‐specific responses to environmental factors and nectar quality differences between collection locations could not be fully assessed in this study due to small sample sizes. Further work is needed to expand the understanding of species‐specific nectar production responses to environmental factors. The longevity of individual flowers could not be assessed in this study due to time constraints and limited field assistance. More detailed characterization of flowering species' nectar resources available within a habitat could meaningfully improve the assessment of species‐specific resource availability over time.

### Species characterization: which species contributes the most sugar and hydration potential when considering variations in floral abundance, nectar volume, and sugar availability per stem?v

Observational studies of Karner Blue flower visitation or feeding suggest this small butterfly is opportunistic when selecting plants for feeding, choosing those with the greatest numbers of flowers or flowering heads and with yellow or white flowers (Grundel *et al*., 2000). Of the 26 taxa in their list of species selected by Karner blues, 15 were in the sites in our survey. Our study characterized 22 flowering species for floral abundance, nectar volume, and sugar concentration per flower. When considering species that occurred in the spring vegetation survey, *Rubus flagellaris* and *Lithospermum canescens* provided the highest sugar concentrations (39.80% and 30.33% Brix, respectively) within the upper range considered optimal for butterflies (Nicolson & Thornburg, 2007). The only species that supplied an optimal butterfly‐feeding sugar concentration was *Fragaria virginiana* (23.67%). In the summer vegetation survey, *Monarda punctata a*nd *Liatris aspera* had the highest sugar concentrations (61.26% and 59.75% Brix, respectively). *Ceanothus americanus* (22.44%) was the only summer species that produced nectar within the optimal range for butterfly feeding within the summer survey. In the spring vegetation survey, *R. flagellaris* and *P. simplex* provided the highest nectar volumes per flower (2.46 and 1.8 μl, respectively). In the summer vegetation survey, *B. tinctoria* and *H. perforatum* had the greatest nectar volumes (2.20 and 1.69 μl, respectively). The abundance of nectar feeders within an ecosystem is often linked to the energy value of resources, calculated through nectar volume and concentration (Roubik, 1989).

We note that nectar is likely important for pollinators to maintain hydration. As nectar is typically dilute, any source of nectar likely serves as an important source of water (Nicolson, 2009), and thus flowers with greater nectar volume offer more water. Previous research suggests that nectivores may also balance their needs for sugar and water, often choosing intermediate sugar concentrations, with suction feeders like butterflies often preferring 30–40% sugar (Kim *et al*., 2011). However, the choice of intermediate concentrations may also be related to the effort of consuming nectar with high viscosity, which varies by the type of mouthparts. Additionally, when butterflies were fed nectar with low sugar concentrations (5%) they preferentially consumed sugar‐rich nectar (30%) when possible (Cahenzli & Erhardt, 2012b), suggesting energy demands overrode hydration needs under these low‐sugar conditions. Overall, more research is needed to understand how nectar volume and concentration influence pollinator hydration and trade‐offs with other requirements.

Floral availability was very important when evaluating a species' total nectar availability. For example, of the 22 species examined *Ceanothus americanus* provided a median sugar concentration per flower but an astounding amount of sugar per stem due to having *c*. 3000 small flowers. For the spring survey, *R. flagellaris* and *L. canescens* remained the highest sugar contributors per stem. From the summer vegetation survey *C. americanus* yielded the most nectar sugar per stem followed by *M. punctata*. The inclusion of species‐specific nectar sugar concentration per flower and total floral availability per plant allowed us to estimate a species' total nectar sugar contribution to a pollinator. Studies have shown that locations with greater nectar resource diversity were more likely to support larger butterfly populations and more diverse bee communities (Williams, 1988; Britten & Riley, 1994; Schultz & Dlugosch, 1999; Potts *et al*., 2004). The nectar resources per stem reported from our study are a valuable contribution to oak savanna habitat assessment, and similar procedures can be applied to future habitat assessments to complete more detailed evaluation.

### Site assessment: how does the quality of habitat nectar resources (sugar density and total per site) vary among sites that differ in Karner blue occupancy or change between the spring and summer seasons?

Our study established a quantitative approach to evaluate oak savanna habitat nectar resources, which revealed temporal differences not observed in a previous vegetation survey for habitat assessment. Nectar resources are typically evaluated through flowering stem density, but this approach can be misleading due to variations in species floral abundance and nectar quality (Schultz & Dlugosch, 1999). We identified significant differences between site nectar sugar availability not previously measured by flowering stem density. Further investigation demonstrated that sites associated with natural Karner blue butterfly occupancy had *c*. 11 times more sugar per quadrat in the spring than REL sites no longer occupied. Three species, *Rubus flagellaris*, *Lithospermum canescens*, and *Coreopsis lanceolata*, were important contributors to this spring sugar availability. As an indicator and umbrella taxon (Shuey, 1997; Fleishman *et al*., 2000; Chan & Packer, 2006) the habitat requirements of the Karner blue butterfly can guide effective oak savanna habitat restoration and pollinator conservation. Our study found it is essential to assess the nectar resources available during both spring and summer to fully understand the resource dynamics between seasons.

The oak savanna vegetation surveys utilized for this study were categorized by Karner blue butterfly occupancy related to a severe regional drought (Walsh, 2017). Sites were categorized as OCC by Karner blue butterflies before and after the drought, no longer occupied after the drought (ROC), REL sites no longer occupied, and high priority RES sites. Walsh (2017) found that OCC sites that successfully supported Karner blue populations through the drought had a greater flowering lupine density, ant entrance density, and heat loads. While Walsh (2017) did not find that nectar plant density had a consistent effect on Karner blue presence, further examination of resource nectar availability may distinguish general limitations to population size (Roulston & Goodell, 2011; Hicks *et al*., 2016).

Studies often correlate butterfly abundance with nectar species abundance (Holl, 1995; Schultz & Dlugosch, 1999). It has been acknowledged that more detailed factors such as metapopulation dynamics (Moilanen & Hanski, 1998; Fred & Brommer, 2003), vegetation height (Ellis, 2003), and nectar quality (Schultz & Dlugosch, 1999) also affect the habitat quality for butterfly species. Estimating resource availability by plant density alone, or even individual flower numbers, fails to capture resource effects from differences in species nectar quality. Nonparametric testing showed no difference in summer flowering stem density between four category comparisons (OCC vs REL; OCC vs RES; FOR vs REL; FOR vs RES). We found a stronger association between the number of stems and sugar per quadrat for Karner OCC sites compared to a weaker association for REL sites, but significant differences in nectar sugar availability among site categories. This varying degree of association between flowering stem density and estimated sugar availability suggests extraneous factors such as habitat management and tree cover (Abella *et al*., 2020) that affect species‐specific floral abundance or nectar quality could influence the accuracy of nectar resource assessment via flowering stem density alone. These results demonstrate that a combination of species‐specific floral abundance and nectar sugar quality can provide an improved assessment of the resources available. Estimating nectar sugar per plant allowed us to evaluate sugar availability at the quadrat and site level to provide new insights into the magnitude and distribution of nectar resources available to oak savanna pollinators. Increased sugar availability provides quantitative improvements in butterfly reproduction. Female butterflies fed nectar containing 20% sugar increased body weight maintenance and total egg production in later oviposition. Male butterflies consuming sugar‐rich diets also benefit from fitness increases by producing more nourishing spermatophores resulting in greater larval hatching mass (Murphy *et al*., 1983). Overall, butterflies consuming adequate quantities of sugar often experience improved fecundity, longevity, and increased fitness over a lifetime (Hill & Pierce, 1989; Mevi‐Schutz & Erhardt, 2005; Bauerfeind & Fischer, 2009; Cahenzli & Erhardt, 2012a).

Increased fecundity associated with greater sugars could help Karner populations. Haack (1993) suggested that a viable population of Karner blue butterflies should have 1000 first‐generation individuals leading to *c*. 3000 second‐generation individuals. If Karner blue adults lay more eggs in the spring, producing larvae feeding in the summer, this could help buffer the effects of lower larval success over the summer when larval resources are insufficient (Patterson *et al*., 2020). Ensuring enough sugar is available in the spring to the first generation may be an important factor to support population viability. Our study also has implications for the conservation of other multivoltine butterflies such as the monarch (*Danaus plexippus*), which arrives as migratory adults in early to mid‐May in the Midwest. Bruce *et al*. (2022) found that both milkweed density and floral species richness were the best predictors of overall adult monarch abundance, with each effect similar in size. A closer examination of nectar resource quality across the seasons might have significant benefits for monarch conservation.

By using sites naturally occupied by the Karner blue butterflies to represent desired oak savanna resource requirements, we were able to show that a greater abundance of spring nectar resources is an important habitat feature. Typically, Karner blue butterflies are released into a new site during the summer as second‐generation adults. Our study shows it is essential to properly assess the nectar resources available during both adult generations to fully understand the resource dynamics between seasons and habitat resource sufficiency. Measuring flowering stem density alone is an incomplete evaluation of resources and can be misleading. A previous study of these nectar sources and sites (Arnold & Michaels, 2017) documented substantial amounts of amino acids in the nectar of some of these species, which can influence lifespan and egg production in some butterfly species (Mevi‐Schutz & Erhardt, 2005) and have been linked to increased egg and larval sizes or increased survival (Bauerfeind & Fischer, 2009; Cahenzli & Erhardt, 2012b). Evaluating both floral abundance and nectar resource quality is necessary to improve habitat restoration and pollinator conservation. The 22 plant species characterized for this study lay the foundation to guide oak savanna species selection and habitat assessment. Measuring flowering stem density is a traditionally preferred method for resource assessment because collecting nectar quality characteristics can be very time intensive (Schultz & Dlugosch, 1999). To reduce future time spent in the field, work should be continued to develop a catalog (trait database) of oak savanna flowering resource characteristics that include floral availability, pollen count, phenology, amino acid concentration, nectar volume, and sugar concentration. These studies should be coupled with new studies on Karner blue foraging preferences to better understand how nectar resources, host plant distribution (Chau *et al*., 2020), and microclimate influence site‐specific population dynamics. Establishing a quantitative approach to nectar resource availability will increase habitat assessment accuracy, enable measurable restoration goals, and further improve habitats for pollinators of this critically imperiled ecosystem.

## Competing interests

None declared.

## Author contributions

MT and HJM planned and designed the research with inputs from RPW and KEMC. MT collected all the data other than stem densities provided by RPW. MT performed all statistical analyses supported by HJM and KEMC. MT wrote the initial draft manuscript. All authors reviewed, edited, and approved the final version.

## Disclaimer

The New Phytologist Foundation remains neutral with regard to jurisdictional claims in maps and in any institutional affiliations.

## Supporting information


**Fig. S1** Non‐metric multidimensional scaling analysis to compare spring flowering community composition.
**Fig. S2** Non‐metric multidimensional scaling analysis to compare summer flowering community composition.
**Fig. S3** Variation in sugar (mg) per stem of species within spring vegetation survey.
**Notes S1** Sample comparisons for *Rubus flagellaris* and *Coreopsis lanceolata* samples from the field and glasshouse.
**Table S1** Species selected for nectar and floral characterization.
**Table S2** Classification of Karner blue conservation sites.
**Table S3** The proportion of non‐flowering/empty quadrats and proportion of plants characterized.
**Table S4** Nectar sampling sites (and abbreviations).
**Table S5** Spearman's rank correlations between nectar composition variables and environmental factors.
**Table S6** General linear model showing the relationship between nectar volume (μl) and sugar concentration per flower with.
**Table S7** General linear model analyses showing the relationship between nectar volume (μl) and sugar availability (mg) per stem with.
**Table S8** Total sample size (*n*) means and SE of sugar (Brix) and nectar volume (μl) per flower.
**Table S9** The number of open flowers per plant when encountered in the field.
**Table S10** Permutational multivariate analysis of variance to test for differences in community composition between site categories.
**Table S11** Wilcoxon/Kruskal–Wallis test to compare the number of flowering stems.
**Table S12** Tukey–Kramer HSD all pairs test to compare the number of flowering stems per 0.5 m^2^ quadrat.
**Table S13** Percent of non‐flowering quadrats for each category.
**Table S14** Wilcoxon/Kruskal–Wallis comparing the number of flowering stems per 0.5 m^2^ quadrat between seasons within each category.
**Table S15** Linear mixed effects models showing the relationship between (a) sugar availability (mg) and (b) nectar volume (μl) per quadrat with category, season, and their interaction.
**Table S16** Generalized linear modeling showing the relationship between site total sugar (g) and total nectar volume (ml) with site category.
**Table S17** Tukey–Kramer HSD all pairs test to compare the number of flowering stems and nectar sugar (mg) per 0.5 m^2^ quadrat.
Please note: Wiley is not responsible for the content or functionality of any Supporting Information supplied by the authors. Any queries (other than missing material) should be directed to the *New Phytologist* Central Office.

## Data Availability

Relevant statistical tables are within the paper and the Supporting Information files. Additional data is available on the Harvard Dataverse data repository using the following citation: Michaels, Helen, 2025, ‘Turner‐Nectar Data’, doi: 10.7910/DVN/BOY6Y1.

## References

[nph70036-bib-0001] Abella SR , Sprow LA , Menard KS , Schetter TA , Brewer LG . 2020. Changes in groundlayer communities with variation in trees, sapling layers, and fires during 34 years of oak savanna restoration. Natural Areas Journal 43: 243–252.

[nph70036-bib-0002] Anderson RC , Fralish JS , Baskin JM , eds. 2007. Savannas, barrens, and rock outcrop plant communities of North America. Cambridge, UK: Cambridge University Press.

[nph70036-bib-0003] Archer CR , Pirk CWW , Carvalheiro LG , Nicolson SW . 2014. Economic and ecological implications of geographic bias in pollinator ecology in the light of pollinator declines. Oikos 123: 401–407.

[nph70036-bib-0004] Arnold PM , Michaels HJ . 2017. Nectar sampling for prairie and oak savanna butterfly restoration. Applications in Plant Sciences 5: 1600148. 10.3732/apps.1600148 PMC5499304 28690931

[nph70036-bib-0005] Baker HG , Baker I . 1975. Studies of nectar‐constitution and pollinator plant coevolution. In: Gilbert LE , Raven PH , eds. Coevolution of animals and plants. Austin, TX, USA: University of Texas Press, 100–140.

[nph70036-bib-0006] Baldock KCR , Goddard MA , Hicks DM , Kunin WE , Mitschunas N , Osgathorpe LM , Potts SG , Robertson KM , Scott AV , Stone GN *et al*. 2015. Where is the UK's pollinator biodiversity? The importance of urban areas for flower‐visiting insects. Proceedings of the Royal Society B: Biological Sciences 282: 20142849. 10.1098/rspb.2014.2849 PMC4345454 25673686

[nph70036-bib-0007] Bauerfeind SS , Fischer K . 2009. Effects of larval starvation and adult diet‐derived amino acids on reproduction in a fruit‐feeding butterfly. Entomologia Experimentalis et Applicata 130: 229–237.

[nph70036-bib-0008] Bergman KO , Kindvall O . 2004. Population viability analysis of the butterfly *Lopinga achine* in a changing landscape in Sweden. Ecography 27: 49–58.

[nph70036-bib-0009] Bertsch A . 1983. Nectar production of *Epilobium angustifolium* L. at different air humidities; nectar sugar in individual flowers and the optimal foraging theory. Oecologia 59: 40–48. 25024144 10.1007/BF00388069

[nph70036-bib-0011] Bolker BM , Brooks ME , Clark CJ , Geange SW , Poulsen JR , Stevens MH , White JS . 2009. Generalized linear mixed models: A practical guide for ecology and evolution. Trends in Ecology & Evolution 24: 127–135. 19185386 10.1016/j.tree.2008.10.008

[nph70036-bib-0012] Bolten AB , Feinsinger P , Baker HG , Baker I . 1979. On the calculation of sugar concentration in flower nectar. Oecologia 41: 301–304. 28309767 10.1007/BF00377434

[nph70036-bib-0013] Britten HG , Riley L . 1994. Nectar source diversity as an indicator of habitat suitability for the endangered Uncompahgre fritillary, *Bolaria acrocnema* (Nymphalidae). Journal of the Lepidopterists' Society 48: 173–179.

[nph70036-bib-0014] Bruce AS , Thogmartin WE , Trosen C , Oberhauser K , Gratton C . 2022. Landscape‐ and local‐level variables affect monarchs in Midwest grasslands. Landscape Ecology 37: 93–108.

[nph70036-bib-0015] Burnham KP , Anderson DR . 2002. Model selection and inference: a practical information‐theoretic approach, 2^nd^ edn. New York, NY, USA: Springer‐Verlag.

[nph70036-bib-0016] Burquez A , Corbet SA . 1991. Do flowers reabsorb nectar? Functional Ecology 5: 369–379.

[nph70036-bib-0017] Cahenzli F , Erhardt A . 2012a. Enhancing offspring quality or quantity? Different ways for using nectar amino acids in female butterflies. Oecologia 169: 1005–1014. 22271202 10.1007/s00442-012-2254-7

[nph70036-bib-0018] Cahenzli F , Erhardt A . 2012b. Nectar sugars enhance fitness in male *Coenonympha pamphilus* butterflies by increasing longevity or realized reproduction. Oikos 121: 1417–1423.

[nph70036-bib-0019] Chan PK , Packer L . 2006. Assessment of potential Karner Blue butterfly (*Lycaeides melissa samuelis*) (family: Lycanidae) reintroduction sites in Ontario. Canada. Restoration Ecology 14: 645–652.

[nph70036-bib-0020] Chau SN , Bristow LV , Grundel R , Hellmann JJ . 2020. Resource segregation at fine spatial scales explains Karner blue butterfly (*Lycaeides melissa samuelis*) distribution. Journal of Insect Conservation 24: 739–749.

[nph70036-bib-0021] Crone EE , Schultz CB . 2022. Host plant limitation of butterflies in highly fragmented landscapes. Theoretical Ecology 15: 165–175.

[nph70036-bib-0022] Dafni A . 1992. Rewards in flowers. In: Rickwood D , Hames BD , eds. Pollination ecology: A practical approach. New York, NY, USA: Oxford University Press, 127–161.

[nph70036-bib-0023] Day M . 2020. Nectar resource quality of oak savanna habitats . MS thesis, Bowling Green State University, Bowling Green, OH, USA.

[nph70036-bib-0024] Ellis S . 2003. Habitat quality and management for the northern brown argus butterfly *Aricia Artaxerxes* (Lepidoptera: Lycaenidae) in North East England. Biological Conservation 113: 285–294.

[nph70036-bib-0025] Farkas Á , Molnár R , Morschhauser T , Hahn I . 2012. Variation in nectar volume and sugar concentration of *Allium ursinum* L. ssp. *ucrainicum* in three habitats. The Scientific World Journal 2012: 138579. 22619588 10.1100/2012/138579 PMC3349315

[nph70036-bib-0026] Fleishman E , Murphy DD , Peter FB . 2000. A new method for selection of umbrella species for conservation planning. Ecological Applications 10: 569–579.

[nph70036-bib-0027] Fred MS , Brommer JE . 2003. Influence of habitat quality and patch size on occupancy and persistence in two populations of the Apollo butterfly (*Parnassius apollo*). Journal of Insect Conservation 7: 85–98.

[nph70036-bib-0028] Freeman CE , Head KC . 1990. Temperature and sucrose composition of floral nectars in *Ipomopsis longiflora* under field conditions. Southwestern Naturalist 35: 423–426.

[nph70036-bib-0029] Gallagher MK , Campbell DR . 2017. Shifts in water availability mediate plant–pollinator interactions. New Phytologist 215: 792–802. 28517023 10.1111/nph.14602

[nph70036-bib-0030] Gilbert FS , Haines N , Dickson K . 1991. Empty flowers. Functional Ecology 5: 29–39.

[nph70036-bib-0031] Grossmann EB , Mladenoff DJ . 2007. Open woodland and savanna decline in a mixed disturbance landscape (1938 to 1998) in the Northwest Wisconsin (USA) Sand Plain. Landscape Ecology 22: 43–55.

[nph70036-bib-0032] Grundel R , Pavlovic NB , Sulzman CL . 2000. Nectar plant selection by the Karner blue butterfly (*Lycaeides melissa samuelis*) at the Indiana Dunes National Lakeshore. The American Midland Naturalist 144: 1–10.

[nph70036-bib-0033] Haack RA . 1993. *The endangered Karner blue butterfly (Lepidoptera: Lycaenidae): biology*, *management considerations*, *and data gaps* . United States Department of Agriculture, Forest Service General Technical Report NC, 83–100.

[nph70036-bib-0034] Hicks DM , Ouvrard P , Baldock KC , Baude M , Goddard MA , Kunin WE , Mitschunas N , Memmott J , Morse H , Nikolitsi M *et al*. 2016. Food for pollinators: quantifying the nectar and pollen resources of urban flower meadows. PLoS ONE 11: e0158117. 27341588 10.1371/journal.pone.0158117 PMC4920406

[nph70036-bib-0035] Hill CJ , Pierce NE . 1989. The effect of adult diet on the biology of butterflies. Oecologia 81: 249–257. 28312544 10.1007/BF00379812

[nph70036-bib-0036] Holl KD . 1995. Nectar resources and their influence on butterfly communities on reclaimed coal surface mines. Restoration Ecology 3: 76–85.

[nph70036-bib-0037] Kato S , Sakai S . 2008. Nectar secretion strategy in three Japanese species: changes in nectar volume and sugar concentration dependent on flower age and flowering order. Botany 86: 337–345.

[nph70036-bib-0038] Kearns CA , Inouye DW . 1993. Techniques for pollination biologists. Niwot, CO, USA: University Press of Colorado.

[nph70036-bib-0039] Kim W , Gilet T , Bush JWM . 2011. Optimal concentrations in nectar feeding. Proceedings of the National Academy of Sciences, USA 108: 16618–16621. 10.1073/pnas.1108642108 PMC3189050 21949358

[nph70036-bib-0040] King RS . 2003. Habitat management: habitat management for the Karner Blue butterfly (*Lycaeides melissa samuelis*) evaluating the short‐term consequences. Ecological Restoration 21: 101–106.

[nph70036-bib-0041] Kocher SD , Williams EH . 2000. The diversity and abundance of North American butterflies vary with habitat disturbance and geography. Journal of Biogeography 27: 785–794.

[nph70036-bib-0042] Kuppler J , Kotowska MM . 2021. A meta‐analysis of responses in floral traits and flower–visitor interactions to water deficit. Global Change Biology 27: 3095–3108. 33774883 10.1111/gcb.15621

[nph70036-bib-0043] Landres PB , Morgan P , Swanson FJ . 1999. Overview of the use of natural variability concepts in managing ecological systems. Ecological Applications 9: 1179–1188.

[nph70036-bib-0044] Leach MK , Givnish TJ . 1999. Gradients in the composition, structure, and diversity of remnant oak savannas in southern Wisconsin. Ecological Monographs 69: 353–374.

[nph70036-bib-0045] Meehan TD , Glassberg J , Gratton C . 2013. Butterfly community structure and landscape composition in agricultural landscapes of the central United States. Journal of Insect Conservation 17: 411–419.

[nph70036-bib-0046] Mevi‐Schutz J , Erhardt A . 2005. Amino acids in nectar enhance butterfly fecundity: a long awaited link. The American Naturalist 164: 411–419. 10.1086/429150 15791533

[nph70036-bib-0047] Mitchell RJ . 2004. Heritability of nectar traits: why do we know so little? Ecology 85: 1527–1533.

[nph70036-bib-0048] Mohr NA , Jay SC . 1990. Nectar production of selected cultivars of *Brassica campestris* L. and *Brassica napus* L. Journal of Apicultural Research 29: 95–100.

[nph70036-bib-0049] Moilanen A , Hanski I . 1998. Metapopulation dynamics: effects of habitat quality and landscape structure. Ecology 79: 2503–2515.

[nph70036-bib-0050] Murphy DD , Launer AE , Ehrlich PR . 1983. The role of adult feeding in egg production and population dynamics of the checkerspot butterfly Euphydryas editha. Oecologia 56: 257–263. 28310203 10.1007/BF00379699

[nph70036-bib-0051] Nicolson SW . 2009. Water homeostasis in bees, with the emphasis on sociality. Journal of Experimental Biology 212: 429–434. 19151218 10.1242/jeb.022343

[nph70036-bib-0052] Nicolson SW , Thornburg RW . 2007. Nectar chemistry. In: Nectaries and nectar. Dordrecht, the Netherlands: Springer Netherlands, 215–263.

[nph70036-bib-0053] Nicolson SW , van Wyk B . 1998. Nectar sugars in Proteaceae: patterns and processes. Australian Journal of Botany 46: 489–504.

[nph70036-bib-0054] Noss RF , LaRoe ET III , Scott JM . 1995. Endangered ecosystems of the United States: a preliminary assessment of loss and degradation . Biological Report 28, National Biological Service, Washington, DC.

[nph70036-bib-0055] Nuzzo V . 1986. Extent and status of Midwest oak savanna: presettlement and 1985. Natural Areas Journal 6: 6–36.

[nph70036-bib-0057] Oksanen J , Blanchet FG , Kindt R , Legendre P , Minchin P , O'Hara B , Simpson G , Solymos P , Stevens H , Wagner H . 2018. vegan: community ecology package . R package v. 2: 4–6.

[nph70036-bib-0058] Olson SD . 1996. The historical occurrence of fire in the Central Hardwoods, with emphasis on southcentral Indiana. Natural Areas Journal 16: 248–256.

[nph70036-bib-0059] Opler P , Malilul V . 1998. A field guide to eastern butterflies. Boston: Houghton Mifflin Co.

[nph70036-bib-0060] Pacini E , Nepi M , Vesprini JL . 2003. Nectar biodiversity: a short review. Plant Systematics and Evolution 238: 7–21.

[nph70036-bib-0061] Pamminger T , Becker R , Himmelreich S , Schneider CW , Bergtold M . 2019. The nectar report: quantitative review of nectar sugar concentrations offered by bee visited flowers in agricultural and non‐agricultural landscapes. PeerJ 7: e6329. 30834180 10.7717/peerj.6329 PMC6397631

[nph70036-bib-0062] Parachnowitsch AL , Manson JS , Sletvold N . 2019. Evolutionary ecology of nectar. Annals of Botany 123: 247–261. 30032269 10.1093/aob/mcy132 PMC6344224

[nph70036-bib-0063] Patterson TA , Grundel R , Dzurisin JD , Knutson RL , Hellmann JJ . 2020. Evidence of an extreme weather‐induced phenological mismatch and a local extirpation of the endangered Karner blue butterfly. Conservation Science and Practice 2: e147.

[nph70036-bib-0064] Pham‐Delègue MH , Mesquida J , Marilleau R , Le Métayer M , Renard M . 1990. Quantitative and qualitative analysis of rapeseed flower nectar (*Brassica napu*s L. var. *oleifera Metzger*). In: VI International symposium on pollination 288, 430–434.

[nph70036-bib-0065] Pickens BA , Root KV . 2008. Factors affecting host‐plant quality and nectar use for the Karner blue butterfly: implications for oak savanna restoration. Natural Areas Journal 28: 210–217.

[nph70036-bib-0066] Potts SG , Vulliamy B , Roberts S , O'Toole C , Dafni A , Ne'eman G , Willmer PG . 2004. Nectar resource diversity organises flower‐visitor community structure. Entomologia Experimentalis et Applicata 113: 103–107.

[nph70036-bib-0067] Roubik DW . 1989. Ecology and natural history of tropical bees. Cambridge, UK: Cambridge University Press. 10.1126/science.248.4958.1026 17745410

[nph70036-bib-0068] Roulston TAH , Goodell K . 2011. The role of resources and risks in regulating wild bee populations. Annual Review of Entomology 56: 293–312. 10.1146/annurev-ento-120709-144802 20822447

[nph70036-bib-0069] Sankaran M , Ratnam J , Hanan JP . 2004. Tree‐grass coexistence in savannas revisited— insights from an examination of assumptions and mechanisms invoked in existing models. Ecology Letters 7: 480–490.

[nph70036-bib-0070] Schultz CB , Dlugosch KM . 1999. Nectar and hostplant scarcity limit populations of an endangered Oregon butterfly. Oecologia 119: 231–238. 28307973 10.1007/s004420050781

[nph70036-bib-0071] Seeley TD . 2009. The wisdom of the hive: the social physiology of honey bee colonies. Cambridge, MA, USA: Harvard University Press.

[nph70036-bib-0072] Shuey JA . 1997. Dancing with fire: ecosystem dynamics, management, and the Karner blue (*Lycaeides melissa samuelis* Nabokov) (Lycaenidae). Journal of the Lepidopterists' Society 51: 263–269.

[nph70036-bib-0073] Stern RA , Gazit S . 1996. Lychee pollination by the honeybee. Journal of the American Society for Horticultural Science 121: 152–157.

[nph70036-bib-0074] Swengel SR , Swengel AB . 1999. Correlations in abundance of grassland song bird sand prairie butterflies. Biological Conservation 90: 1–11.

[nph70036-bib-0075] Swetnam TW , Allen CD , Betancourt JL . 1999. Applied historical ecology: using the past to manage for the future. Ecological Applications 9: 1189–1206.

[nph70036-bib-0076] Tew NE , Memmott J , Vaughan IP , Bird S , Stone GN , Potts SG , Baldock KCR . 2021. Quantifying nectar production by flowering plants in urban and rural landscapes. Journal of Ecology 109: 1747–1757.

[nph70036-bib-0078] U.S. Fish and Wildlife Service . 1992. Endangered and threatened wildlife and plants; determination of endangered status for the Karner blue butterfly. Final rule. Federal Register 57: 59236–59244.

[nph70036-bib-0079] Villarreal AG , Freeman CE . 1990. Effects of temperature and water stress on some floral nectar characteristics in *Ipomopsis longiflora* (Polemoniaceae) under controlled conditions. Botanical Gazette 151: 5–9.

[nph70036-bib-0081] Walsh RP . 2017. Microclimate and biotic interactions affect Karner blue butterfly occupancy and persistence in managed oak savanna habitats. Journal of Insect Conservation 21: 219–230.

[nph70036-bib-0082] Williams EH . 1988. Habitat and range of *Euphydryas gillettii* (Nymphalidae). Journal of the Lepidopterists' Sociekkliukty 42: 37–45.

[nph70036-bib-0083] Willmer PG . 2011. Reward 2: the biology of nectar. In: Pollination and floral ecology. Princeton, NJ, USA: Princeton University Press, 190–220.

[nph70036-bib-0084] Wyatt R , Broyles SB , Derda GS . 1992. Environmental influences on nectar production in milkweeds (*Asclepias syriaca* and *A. exaltata*). American Journal of Botany 79: 636–642.

