# Supplementary material for: Nectar in oak savannas: implications for butterfly conservation
Source: New Phytol. 2025 Mar 9;246(3):1390–402. doi: 10.1111/nph.70036 (PMC11982782; doi:10.1111/nph.70036)
Supplement: Supplementary file 1 — Fig. S1 Non‐metric multidimensional scaling analysis to compare spring flowering community composition. Fig. S2 Non‐metric multidimensional scaling analysis to compare summer flowering community composition. Fig. S3 Variation in sugar (mg) per stem of species within spring vegetation survey. Notes S1 Sample comparisons for Rubus flagellaris and Coreopsis lanceolata samples from the field and glasshouse. Table S1 Species selected for nectar and floral characterization. Table S2 Classification of Karner blue conservation sites. Table S3 The proportion of non‐flowering/empty quadrats and proportion of plants characterized. Table S4 Nectar sampling sites (and abbreviations). Table S5 Spearman's rank correlations between nectar composition variables and environmental factors. Table S6 General linear model showing the relationship between nectar volume (μl) and sugar concentration per flower with. Table S7 General linear model analyses showing the relationship between nectar volume (μl) and sugar availability (mg) per stem with. Table S8 Total sample size (n) means and SE of sugar (Brix) and nectar volume (μl) per flower. Table S9 The number of open flowers per plant when encountered in the field. Table S10 Permutational multivariate analysis of variance to test for differences in community composition between site categories. Table S11 Wilcoxon/Kruskal–Wallis test to compare the number of flowering stems. Table S12 Tukey–Kramer HSD all pairs test to compare the number of flowering stems per 0.5 m2 quadrat. Table S13 Percent of non‐flowering quadrats for each category. Table S14 Wilcoxon/Kruskal–Wallis comparing the number of flowering stems per 0.5 m2 quadrat between seasons within each category. Table S15 Linear mixed effects models showing the relationship between (a) sugar availability (mg) and (b) nectar volume (μl) per quadrat with category, season, and their interaction. Table S16 Generalized linear modeling showing the relationship between site total sugar (g) and [file NPH-246-1390-s001.docx]

## *New Phytologist* Supporting Information

Article title: Nectar in Oak Savannas: Implications for Butterfly Conservation

Authors: Meigan Turner, Kevin E. McCluney, Ryan P. Walsh, Helen J. Michaels

Article acceptance date: 4 February 2025.

The following Supporting Information is available for this article (Short legends):

**Fig. S1** Non-metric multidimensional scaling analysis to compare spring flowering community composition

**Fig. S2** Non-metric multidimensional scaling analysis to compare summer flowering community composition

**Fig. S3** Variation in sugar (mg) per stem of species within spring vegetation survey.

**Table S1** Species selected for nectar and floral characterization

**Table S2** Classification of Karner blue conservation sites

**Table S3** The proportion of non-flowering/empty quadrats and proportion of plants characterized

**Table S4** Nectar sampling sites (and abbreviations)

**Table S5** Spearman’s rank correlations between nectar composition variables and environmental factors

**Table S6** General linear model showing the relationship between **(a)** nectar volume (µL) and **(b)** sugar concentration per flower with

**Table S7** General linear model analyses showing the relationship between **(a)** nectar volume (µL) and **(b)** sugar availability (mg) per stem with

**Table S8** Total sample size (N) Means and standard errors (in parentheses) of sugar (Brix) and nectar volume (µL) per flower

**Table S9** The number of open flowers per plant when encountered in the field.

**Table S10** Permutational multivariate analysis of variance (PERMANOVA) to test for differences in community composition between site categories

**Table S11** Wilcoxon/Kruskal-Wallis test to compare the number of flowering stems

**Table S12** Tukey-Kramer HSD all pairs test to compare the number of flowering stems per 0.5 m^2^ quadrat

**Table S13** Percent of non-flowering quadrats for each category

**Table S14** Wilcoxon/Kruskal-Wallis comparing the number of flowering stems per 0.5 m^2^ quadrat between seasons within each category

**Table S15** Linear mixed effects models showing the relationship between (a) sugar availability (mg) and (b) nectar volume (µL) per quadrat with

**Table S16** Generalized linear modeling showing the relationship between site (a) total sugar (g) and (b) total nectar volume (mL) with site category

**Table S17** Tukey-Kramer HSD all pairs test to compare the number of flowering stems and nectar sugar (mg) per 0.5 m^2^ quadrat

**Notes S1** Sample comparisons for *Rubus flagellaris* and *Coreopsis lanceolata* samples from the field and greenhouse

Full legends and Figures/Tables

**Fig. S1** Non-metric multidimensional scaling analysis to compare spring flowering community composition (n = 36 species) between site categories; currently occupied (Occ), formerly occupied (For), release (Rel), restoration (Res). Site categories were found to have significantly different community composition predicted by species stem abundance (p = 0.03). Occupied and formerly occupied sites oriented away from release and restoration sites.


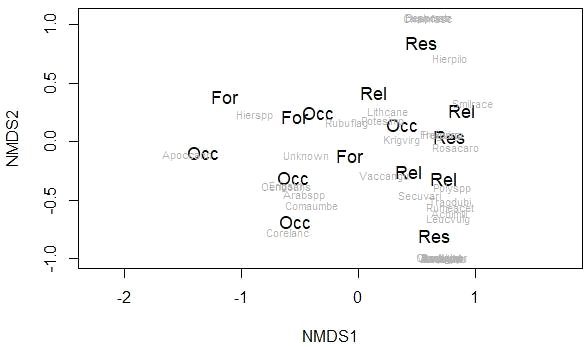


**Fig. S2** Non-metric multidimensional scaling analysis to compare summer flowering community composition (n = 45 species) between site categories; currently occupied (Occ), formerly occupied (For), release (Rel), restoration (Res). Site categories were found to have a non-significant trend for occupied and formerly occupied sites to be oriented away from release and restoration sites (p = 0.09).


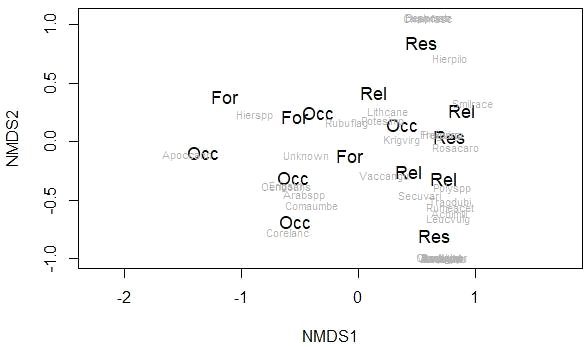


**Fig. S3** Variation in sugar (mg) per stem of species within spring vegetation survey. The sugar concentration (Brix**)** of each nectar sample was converted to milligrams and applied to the mean number of flowers per stem for each species. Error bars represent the estimated range of total sugar per stem within that species while dots represent outliers.


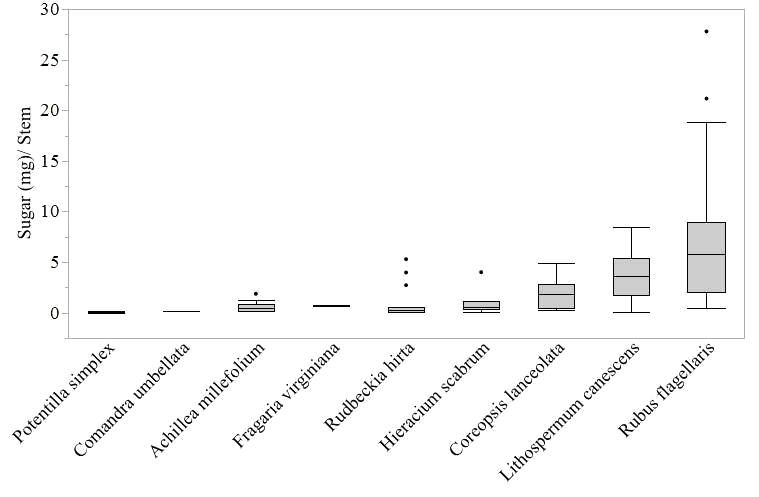


**Table S1** Species selected for nectar and floral characterization, presence during spring and summer surveys or both; number of sites where a species occurred during specified season; and their abundance (mean number of stems in the ground) within a site during each season. When appearing in both spring and summer surveys, sites and mean number of stems are listed as spring followed by summer. Stem data was collected by Walsh (2017). Twenty-five species from that survey were not characterized due to low densities across few sites or were excluded when recorded as ‘Unknown’ or not identified to species. Records of *Hieracium* spp. were an exception because of its presence in nine of the 15 sites at a relatively high frequency (1-370 stems). We sampled nectar of *Hieracium scabrum* (Rough Hawkweed), a native species found in prairie savannas, open woodlands, and clearings with a recorded presence throughout Allegan State Game Area, MI and the Oak Openings Region of OH (Voss, 1996; U.S. Department of Agriculture, 2019) to represent all samples of *Hieracium* spp.

| **Species Name** | **Common Name** | **Brood** | **# Sites** | **Avg. #**  **Stems** |
| --- | --- | --- | --- | --- |
| *Achillea millefolium* L. | Yarrow | Both | 2;4 | 13; 8 |
| *Asclepias tuberosa* L. | Butterfly milkweed | Summer | 2 | 2.5 |
| *Baptisia tinctoria* L. R. Br. | Yellow indigo | Summer | 4 | 7.5 |
| *Ceanothus americanus* L. | New jersey tea | Summer | 2 | 45 |
| *Comandra umbellata* L. Nutt. | Bastard toadflax | Both | 3;2 | 6;3 |
| *Coreopsis lanceolata* L. | Lance-leaved coreopsis | Both | 2; 5 | 5; 11 |
| *Dianthus armeria* L. | Deptford pink | Summer | 4 | 16 |
| *Euphorbia corollata* L. | Flowering spurge | Both | 1; 11 | 8; 17 |
| *Fragaria virginiana* Duchesne | Virginia strawberry | Spring | 5 | 9 |
| *Helianthus divaricatus* L. | Woodland sunflower | Summer | 4 | 50 |
| *Hieracium pilosella* L. | Mouse-ear hawkweed | Spring | 2 | 1 |
| *Hieracium spp.* | Hawkweed spp. | Both | 7; 2 | 125; 8 |
| *Hypericum perforatum* L. | Perforate St. John's wort | Summer | 2 | 3 |
| *Krigia virginica* L. | Dwarf dandelion | Both | 6; 1 | 14; 1 |
| *Lespedeza capitata* Michx. | Round-headed bush clover | Summer | 6 | 33 |
| *Liatris aspera* Michx. | Rough blazing star | Summer | 5 | 4 |
| *Lithospermum canescens* Michx. | Hoary puccoon | Spring | 3 | 5 |
| *Monarda fistulosa* L. | Wild bergamot | Summer | 2 | 22 |
| *Monarda punctata* L. | Dotted horsemint | Summer | 4 | 39 |
| *Potentilla simplex* Michx. | Cinquefoil | Both | 11; 1 | 18; 1 |
| *Rosa carolina* L. | Carolina rose | Spring | 3 | 22 |
| *Rubus flagellaris* Willd. | Dewberry | Spring | 10 | 57 |
| *Rudbeckia hirta* L. | Black-eyed susan | Both | 1; 2 | 2; 7 |

**Table S2** Classification of Karner blue conservation sites. To select oak savanna species for nectar characterization, we utilized vegetation surveys of 15 oak savannas conducted by Walsh (2017) within the Oak Openings Region of northwest Ohio (n = 8) and the Allegan State Game Area in western Michigan (n = 7). Because this vegetation survey was designed to better understand habitat characteristics conducive to Karner blue butterfly survival, sites had been chosen based on Karner blue presence (1) Currently Occupied (OCC); before and after a drought in 2012, (2) Formerly Occupied (FOR); no longer occupied after the drought, (3) Previous Release (REL); previous release sites no longer occupied, and (4) Priority Restoration(RES); high priority restoration sites for future releases. All sites formerly occupied by the Karner blue were naturally occurring populations and not reintroduced by managers. Priority restoration sites were not associated with Karner blue occupancy but were assessed for oak savanna health and potential to become a future release location. Surveys recorded spring (late May/early June) and summer (early July) density of flowering plants defined as the number of flowering stems in the ground per m2. State where each site is located (in parenthesis) is followed by the number of quadrats sampled during each season as an indicator of habitat size.

| (1) Currently (2)  Occupied | Formerly (3)  Occupied | Previous (4)  Release | Priority  Restoration |  |
| --- | --- | --- | --- | --- |
| O1 (MI), 24 | F1 (MI), 24 | P1 (OH), 24 | R1 (OH), 72 | |
| O2 (MI), 30 | F2 (MI), 24 | P2 (OH), 42 | R2 (OH), 30 | |
| O3 (MI), 18 | F3 (MI), 30 | P3 (OH), 72 | R3 (OH), 60 | |
| O4 (MI), 24  O5 (OH), 30 | P4 (OH), 24 | |  | |

**Table S3** The proportion of non-flowering/empty quadrats and proportion of plants characterized for floral and nectar attributes within each site and season. P3, R2, R3 (spring), and P1 (spring and summer) had high densities of a species not frequently found in other sites. O4 and R3 (summer) had a high density of species unidentified in Walsh (2017). R1 (spring and summer) had the greatest species richness of all sites surveyed for both seasons, making assessment of nectar resources there likely to be an underestimate. Six of the 15 sites had greater than 90% of stems characterized for both spring and summer surveys. An additional three sites had 70-90% of the stems characterized within each season.

|  | **Spring** | | **Summer** | |
| --- | --- | --- | --- | --- |
| **Site** | **% Empty**  **Quadrats** | **% Stems** **Characterized** | **% Empty**  **Quadrats** | **% Stems Characterized** |
| O1 | 37.50 | 71.83 | 50.00 | 75.00 |
| O2 | 30.00 | 92.82 | 23.33 | 72.41 |
| O3 | 66.67 | 96.97 | 88.89 | 100.00 |
| O4 | 66.67 | 100.00 | 83.33 | 16.00 |
| O5 | 20.00 | 91.20 | 80.00 | 91.67 |
| F1 | 58.33 | 93.22 | 75.00 | 71.15 |
| F2 | 70.83 | 98.65 | 54.17 | 100.00 |
| F3 | 40.00 | 100.00 | 30.00 | 100.00 |
| P1 | 41.67 | 43.66 | 20.83 | 58.65 |
| P2 | 45.24 | 95.74 | 35.71 | 91.27 |
| P3 | 27.78 | 33.56 | 56.94 | 100.00 |
| P4 | 54.17 | 100.00 | 58.33 | 100.00 |
| R1 | 25.00 | 40.00 | 31.67 | 40.55 |
| R2 | 20.00 | 41.27 | 36.67 | 100.00 |
| R3 | 86.67 | 55.56 | 10.00 | 54.68 |

**Table S4** Nectar sampling sites (and abbreviations); Sites from Lucas Co., OH: Bond (B), Cactus Hill (CH), Julia’s Savanna (JS), Oak Dune (OD), South Piel (SP), and Wahl (W) from Kitty Todd Nature Conservancy; Blue Creek Seed Nursery (BC), Corridor 15 (C15), Flying Tigers (TMG), Jeffers (TMJ), Lark Sparrow Meadow (TML), Parkway (TPK), and Wabash (TMW) from the Toledo Metroparks; Central (LCC) and Entrance (LCE) from Lou Campbell State Nature Preserve; Southview Savanna (SS) from the Olander Park System; Visitor’s Service (VS) from the Toledo Zoo; Helen’s Yard (HY); and Crissey Road (CR); sites in Wood Co.: Rudolph Savanna (RS) from the Wood County Park District; Greenhouse (GH), Ecological Research Station (ERS), and Poe Prairie (PP) at Bowling Green State University; Wintergarden (WG) from the Bowling Green Parks and Recreation. N = total locations sampled. Number in parenthesis is the number of samples from a location. Asterisk indicates species nectar composition characterized by Arnold and Michaels (2017).

**Table S5** Spearman’s rank correlations between nectar composition variables and environmental factors. Asterisks indicate statistical significance at the α = 0.05 level.

| **Variables** |  |  | **Direction of Correlation** | **Spearman’s**  **Coefficient** | **p - value** |
| --- | --- | --- | --- | --- | --- |
| Volume | x | Canopy Cover | + | 0.10 | 0.34 |
| Volume | x | Relative Humidity | + | 0.31 | 0.0005* |
| Volume | x | Soil Moisture | + | 0.34 | 0.0008* |
| Volume | x | Temperature | - | 0.34 | < 0.0001* |
| Volume | x | Sugar | + | 0.009 | 0.89 |
| Sugar | x | Canopy Cover | - | 0.19 | 0.07 |
| Sugar | x | Relative Humidity | - | 0.10 | 0.26 |
| Sugar | x | Soil Moisture | + | 0.07 | 0.89 |
| Sugar | x | Temperature | - | 0.08 | 0.38 |

**Table S6** General linear model showing the relationship between **(a)** nectar volume (µL) and **(b)** sugar concentration (Brix) per flower with species identity, canopy cover (%), relative humidity (%), temperature (°F), and soil moisture (%). The best model is listed at the bottom and identified in bold. ∆AICc compares all models to the best model. All models were significant (p < 0.0001). Parameter estimate of species identity was averaged (mean) across all species. Statistical significance set at the α = 0.05 level.

1. Nectar Volume per Flower

| **Dependent**  **Variable** | **Fixed Effect** | **Estimate** | **p-value** | **Adj R^2^** | **AICc** | **∆AICc** |
| --- | --- | --- | --- | --- | --- | --- |
| Log10 (µL per flower) | Species Identity | 0.10 | < 0.0001 | 0.78 | 132.64 | 4.17 |
|  | Relative Humidity | 0.01 | 0.001 |  |  |  |
|  | Canopy Cover | 0.004 | 0.12 |  |  |  |
|  | Soil Moisture | 0.004 | 0.25 |  |  |  |
|  | Temperature | -0.004 | 0.67 |  |  |  |
| **Dependent**  **Variable** | **Fixed Effect** | **Estimate** | **p-value** | **Adj R^2^** | **AICc** | **∆AICc** |
| Log 10 (µL per flower) | Species Identity | 0.08 | < 0.0001 | 0.77 | 157.83 | 29.36 |
|  | Relative Humidity | 0.02 | < 0.0001 |  |  |  |
| **Dependent**  **Variable** | **Fixed Effect** | **Estimate** | **p-value** | **Adj R^2^** | **AICc** | **∆AICc** |
| **Log 10**  **(µL per flower)** | Species Identity | 0.10 | < 0.0001 | 0.78 | 128.47 | -- |
|  | Relative Humidity | 0.02 | < 0.0001 |  |  |  |
|  | Canopy Cover | 0.003 | 0.15 |  |  |  |

1. Sugar Concentration per Flower

| **Dependent**  **Variable** | **Fixed Effect** | **Estimate** | **p-value** | **Adj R^2^** | **AICc** | **∆AICc** |
| --- | --- | --- | --- | --- | --- | --- |
| Log10  (Brix per flower) | Species Identity | 0.04 | < 0.0001 | 0.52 | 99.16 | 5.41 |
|  | Canopy Cover | -0.01 | 0.002 |  |  |  |
|  | Relative Humidity | -0.01 | 0.01 |  |  |  |
|  | Soil Moisture | 0.002 | 0.49 |  |  |  |
|  | Temperature | -0.003 | 0.74 |  |  |  |
| **Dependent**  **Variable** | **Fixed Effect** | **Estimate** | **p-value** | **Adj R^2^** | **AICc** | **∆AICc** |
| Log10  (Brix per flower) | Species Identity | 0.02 | < 0.0001 | 0.49 | 98.92 | 5.17 |
|  | Canopy Cover | -0.01 | 0.005 |  |  |  |
| **Dependent**  **Variable** | **Fixed Effect** | **Estimate** | **p-value** | **Adj R^2^** | **AICc** | **∆AICc** |
| **Log10**  **(Brix per flower)** | Species Identity | 0.02 | < 0.0001 | 0.52 | 93.75 | -- |
|  | Canopy Cover | -0.006 | 0.001 |  |  |  |
|  | Relative Humidity | -0.008 | 0.01 |  |  |  |

**Table S7** General linear model analyses showing the relationship between **(a)** nectar volume (µL) and **(b)** sugar availability (mg) per stem with species identity, canopy cover (%), relative humidity (%), temperature (°F), and soil moisture (%). The best model is listed at the bottom and identified in bold. ∆AICc compares all models to the best model. All models were significant (p < 0.0001). Parameter estimate of species identity was averaged (mean) across all species. Statistical significance set at the α = 0.05 level.

1. Nectar Volume per Stem

| **Dependent**  **Variable** | **Fixed Effect** | **Estimate** | **p-value** | **Adj R^2^** | **AICc** | **∆AICc** |
| --- | --- | --- | --- | --- | --- | --- |
| Log10  (µL per stem) | Species Identity | 0.01 | < 0.0001 | 0.55 | 132.64 | 4.17 |
|  | Relative Humidity | 0.02 | 0.001 |  |  |  |
|  | Canopy Cover | 0.004 | 0.11 |  |  |  |
|  | Soil Moisture | 0.004 | 0.25 |  |  |  |
|  | Temperature | -0.004 | 0.67 |  |  |  |
| **Dependent**  **Variable** | **Fixed Effect** | **Estimate** | **p-value** | **Adj R^2^** | **AICc** | **∆AICc** |
| Log10  (µL per stem) | Species Identity | 0.001 | < 0.0001 | 0.58 | 157.83 | 29.36 |
|  | Relative Humidity | 0.02 | < 0.0001 |  |  |  |
| **Dependent**  **Variable** | **Fixed Effect** | **Estimate** | **p-value** | **Adj R^2^** | **AICc** | **∆AICc** |
| **Log10**  **(µL per stem)** | Species Identity | 0.10 | < 0.0001 | 0.55 | 128.47 | -- |
|  | Relative Humidity | 0.02 | < 0.0001 |  |  |  |
|  | Canopy Cover | 0.01 | 0.15 |  |  |  |

1. Sugar Availability per Stem

| **Dependent**  **Variable** | **Fixed Effect** | **Estimate** | **p-value** | **Adj R^2^** | **AICc** | **ΔAICc** |
| --- | --- | --- | --- | --- | --- | --- |
| Log 10  (Sugar (mg) per stem) | Species Identity | -0.08 | < 0.0001 | 0.73 | 151.39 | 5.08 |
|  | Canopy Cover | -0.004 | 0.15 |  |  |  |
|  | Relative Humidity | 0.005 | 0.33 |  |  |  |
|  | Soil Moisture | 0.004 | 0.31 |  |  |  |
|  | Temperature | -0.003 | 0.80 |  |  |  |
| **Dependent**  **Variable** | **Fixed Effect** | **Estimate** | **p-value** | **Adj R^2^** | **AICc** | **∆AICc** |
| Log10  (Sugar (mg) per stem) | Species Identity | -0.07 | < 0.0001 | 0.73 | 146.58 | 0.27 |
|  | Canopy Cover | -0.004 | 0.10 |  |  |  |
|  | Relative Humidity | 0.006 | 0.14 |  |  |  |
| **Dependent**  **Variable** | **Fixed Effect** | **Estimate** | **p-value** | **Adj R^2^** | **AICc** | **∆AICc** |
| **Log10**  **(Sugar (mg) per stem)** | Species Identity | -0.07 | < 0.0001 | 0.72 | 146.31 | -- |
|  | Canopy Cover | -0.004 | 0.05 |  |  |  |

**Table S8** Total sample size (N) Means and standard errors (in parentheses) of sugar (Brix) and nectar volume (µL) per flower for each taxon. N = total sample size across all locations. Asterisk indicates species nectar composition characterized by Arnold and Michaels (2017).

| **Scientific name** | **Common name** | **N** | **Mean Sugar (mg)** | **Mean Volume (**µl**)** |
| --- | --- | --- | --- | --- |
| *Achillea millefolium* L. | Yarrow | 15 | 5.33 (1.21) | 0.17 (0.075) |
| *Asclepias tuberosa* L.* | Butterfly milkweed | 30/31 | 57.23 (17.57) | 0.15 (0.08) |
| *Baptisia tinctoria* L. R. Br. | Yellow indigo | 9 | 14.56 (5.46) | 2.20 (0.50) |
| *Ceanothus americanus* L. | New jersey tea | 24 | 22.44 (2.17) | 0.15 (0.030) |
| *Comandra umbellata* L. Nutt. | Bastard toadflax | 1 | 30 (--) | 0.02 (--) |
| *Coreopsis lanceolata* L. | Lance-leaved coreopsis | 14 | 28.39 (3.85) | 0.064 (0.019) |
| *Coreopsis lanceolata* L.* | Lance-leaved coreopsis | 5/7 | 33.60 (6.65) | 0.07 (0.032) |
| *Dianthus armeria* L. | Deptford pink | 7 | 12.64 (3.50) | 0.38 (0.23) |
| *Euphorbia corollata* L. | Flowering spurge | 5 | 6.50 (2.24) | 0.048 (0.009) |
| *Fragaria virginiana* Duchesne | Virginia strawberry | 3 | 23.67 (3.67) | 0.53 (0.065) |
| *Helianthus divaricatus* L.* | Woodland sunflower | 19 | 43.61 (4.08) | 0.06 (0.007) |
| *Hieracium scabrum* Michx. | Rough hawkweed | 7 | 13.79 (2.77) | 0.041 (0.013) |
| *Hypericum perforatum* L. | St. Johnswort | 8 | 3.06 (0.99) | 1.69 (0.52) |
| *Lespedeza capitata* Michx.* | Round-headed bush clover | 12 | 32.67 (3.92) | 0.45 (0.108) |
| *Liatris aspera* Michx. * | Rough blazing star | 16 | 59.75 (3.29) | 0.12 (0.02) |
| *Lithospermum canescens* Michx. | Hoary puccoon | 22 | 30.33 (4.02) | 1.05 (0.14) |
| *Monarda fistulosa* L.* | Wild bergamot | 13 | 56.12 (3.8) | 0.30 (0.07) |
| *Monarda punctata* L.* | Dotted horsemint | 20 | 61.28 (2.93) | 0.27 (0.03) |
| *Potentilla simplex* Michx. | Cinquefoil | 5 | 12.08 (7.88) | 1.80 (0.82) |
| *Rosa carolina* L. | Pasture rose | 1 | 6 (--) | 1.00 (--) |
| *Rubus flagellaris* Willd. | Dewberry | 52 | 39.80 (3.07) | 1.83 (0.23) |
| *Rudbeckia hirta* L. | Black-eyed susan | 16 | 6.78 (2.30) | 0.068 (0.028) |

**Table S9** The number of open flowers per stem (~per plant) when encountered in the field. Sample size (Nstems) refers to the total number of stems in the ground counted for floral availability across all sampling. Means and standard errors of sugar concentration (Brix) and nectar volume per stem.

| **Scientific Name** | **# Flowers (N_stems_)** | **Volume (SE)** | **Sugar (SE)** |
| --- | --- | --- | --- |
| *Achillea millefolium* L. | 114.13 (108) | 18.99 (8.59) | 0.58 (0.12) |
| *Asclepias tuberosa* L. | 63.84 (107) | 9.51(1.17) | 7.68 (1.33) |
| *Baptisia tinctoria* L. R. Br. | 25.34 (50) | 55.75 (12.62) | 10.60 (4.74) |
| *Ceanothus americanus* L. | 3,000.78 (55) | 442.78 (88.96) | 134.20 (36.97) |
| *Comandra umbellata* L. Nutt. | 24.23 (39) | 0.48 (--) | 0.16 (--) |
| *Coreopsis lanceolata* L. | 151.15 (31) | 9.87 (2.44) | 2.04 (0.34) |
| *Dianthus armeria* L. | 2.57 (48) | 0.98 (0.59) | 0.26 (0.21) |
| *Euphorbia corollata* L. | 48.56 (112) | 2.33 (0.42) | 0.15 (0.05) |
| *Fragaria virginiana* Duchesne | 5.06 (111) | 2.70 (0.33) | 0.68 (0.03) |
| *Helianthus divaricatus* L. | 31.37 (195) | 1.77 (0.21) | 0.98 (0.17) |
| *Hieracium scabrum* Michx. | 140.87 (95) | 5.72 (1.77) | 1.08 (0.51) |
| *Hypericum perforatum* L. | 5.90 (134) | 9.96 (3.10) | 0.22 (0.06) |
| *Krigia virginiana* L. | 20.88 (131) | 0 | 0 |
| *Lespedeza capitata* Michx. | 92.55 (86) | 41.81 (10.03) | 12.72 (2.06) |
| *Liatris aspera* Michx. | 184.49 (107) | 23.34 (4.03) | 17.53 (3.21) |
| *Lithospermum canescens* Michx. | 11.01 (365) | 11.61 (1.52) | 3.69 (0.50) |
| *Monarda fistulosa* L. | 179.87 (121) | 53.39 (12.26) | 35.61 (7.11) |
| *Monarda punctata* L. | 180.87 (108) | 51.89 (5.28) | 40.84 (5.20) |
| *Potentilla simplex* Michx. | 1.03 (265) | 1.86 (0.85) | 0.06 (0.03) |
| *Rosa carolina* L. | 1.64 (140) | 1.64 (--) | 0.10 (--) |
| *Rubus flagellaris* Willd. | 9.03 (92) | 16.53 (2.12) | 6.90 (0.83) |
| *Rudbeckia hirta* L. | 254.93 (107) | 17.37 (7.10) | 0.92 (0.40) |

**Table S10** Permutational multivariate analysis of variance (PERMANOVA) to test for differences in community composition between site categories within the (a) spring and (b) summer vegetation survey.

a) Spring

|  | **Sum Sq** | **Mean Sq** | **Df** | **F** | **Pr(>F)** |
| --- | --- | --- | --- | --- | --- |
| Category | 1.58 | 0.53 | 3 | 1.88 | 0.03 |
| Residuals | 3.09 | 0.28 | 11 |  |  |
| Total | 4.67 |  | 14 |  |  |
| b) Summer |  |  |  |  |  |
|  | **Sum Sq** | **Mean Sq** | **Df** | **F** | **Pr(>F)** |
| Category | 1.45 | 0.48 | 3 | 1.37 | 0.09 |
| Residuals | 3.88 | 0.35 | 11 |  |  |
| Total | 5.32 |  | 14 |  |  |

**Table S11** Wilcoxon/Kruskal-Wallis test to compare the number of flowering stems per 0.5 m^2^ quadrat between categories within the spring and summer vegetation surveys. Analysis includes quadrats void of flowering stems. Parenthesis indicates standard deviation. Asterisks indicate statistical significance at the α = 0.05 level.

| **Season** | **Category** | **Mean** | **DF** | ***X*2** | **Prob > *X*^2^** |
| --- | --- | --- | --- | --- | --- |
| Spring | Occupied | 4.86 (9.72) | 3 | 3.67 | 0.30 |
|  | Former | 4.79 (8.78) |  |  |  |
|  | Release | 3.82 (6.30) |  |  |  |
|  | Restoration | 3.63 (4.95) |  |  |  |
| Summer | Occupied | 1.28 (2.59) | 3 | 72.23 | < 0.0001* |
|  | Former | 1.63 (3.01) |  |  |  |
|  | Release | 2.14 (3.28) |  |  |  |
|  | Restoration | 3.46 (5.73) |  |  |  |

**Table S12** Tukey-Kramer HSD all pairs test to compare the number of flowering stems per 0.5 m^2^ quadrat between categories within the spring and summer vegetation surveys. Analysis includes quadrats void of flowering stems. Data received a Log10 transformation. The difference in mean shows the actual absolute difference in the means minus the honest significant difference (HSD).

| **Season** | **Category** | **Δ Mean** | **p-value** |
| --- | --- | --- | --- |
| Spring | Occupied - Former | 0.07 | 0.77 |
|  | Occupied - Release | 0.004 | 0.99 |
|  | Occupied - Restoration | 0.02 | 0.99 |
|  | Former - Release | 0.08 | 0.71 |
|  | Former - Restoration | 0.10 | 0.57 |
|  | Release - Restoration | 0.02 | 0.99 |
| Summer | Occupied - Former | 0.07 | 0.60 |
|  | Occupied - Release | 0.15 | 0.007* |
|  | Occupied - Restoration | 0.36 | < 0.0001* |
|  | Former - Release | 0.08 | 0.44 |
|  | Former – Restoration | 0.29 | < 0.0001* |
|  | Release - Restoration | 0.21 | < 0.0001* |

**Table S13** Percent of non-flowering quadrats for each category appearing with spring and summer vegetation survey.

| **Category** | **Spring** | **Summer** |
| --- | --- | --- |
| Currently Occupied | 41.27% | 62.70% |
| Formerly Occupied | 55.13% | 51.28% |
| Previous Release | 38.27% | 46.91% |
| Priority Restoration | 52.47% | 22.22% |

**Table S14** Wilcoxon/Kruskal-Wallis comparing the number of flowering stems per 0.5 m^2^ quadrat between seasons within each category. Analysis includes quadrats void of flowering stems. Parenthesis indicates standard deviation. Asterisks indicate statistical significance at the α = 0.05 level.

| **Category** | **Category** | **Mean** | **DF** | ***X*2** | **Prob > *X*^2^** |
| --- | --- | --- | --- | --- | --- |
| Occupied | Spring | 4.86 (9.72) | 1 | 22.89 | < 0.0001* |
|  | Summer | 1.28 (2.59) |  |  |  |
| Former | Spring | 4.79 (8.78) | 1 | 0.84 | 0.36 |
|  | Summer | 1.63 (3.01) |  |  |  |
| Release | Spring | 3.82 (6.30) | 1 | 8.57 | 0.003* |
|  | Summer | 2.14 (3.28) |  |  |  |
| Restoration | Spring | 3.63 (4.95) | 1 | 0.44 | 0.51 |
|  | Summer | 3.46 (5.73) |  |  |  |

**Table S15** Linear mixed effects models showing the relationship between (a) sugar availability (mg) and (b) nectar volume (µL) per quadrat with category, season (spring and summer), the interaction between category and season as fixed effects, and site as a random effect. Parameter estimates were averaged (mean) across all interaction effects. Quadrat containing < 90% characterized stems were removed from all models. Statistical significance set at the α = 0.05 level.

1. Sugar availability per quadrat

| **Dependent**  **Variable** | **Fixed Effect** | **Estimate** | **p-value** | **Adj R^2^** | **Model** **p-value** |
| --- | --- | --- | --- | --- | --- |
| Log10  (sugar/ quadrat +1) | Category*  Season | 0.10 | < 0.0001 | 0.16 | < 0.0001 |
|  | Season | -0.05 | 0.03 |  |  |
|  | Category | -0.03 | 0.75 |  |  |
|  | Total Quadrats | 0.01 | 0.21 |  |  |
|  | **Random Effect** | **Variance Ratio** | **DF** |  |  |
|  | Site | 0.08 | 14 |  |  |

1. Nectar volume per quadrat

| **Dependent**  **Variable** | **Fixed Effect** | **Estimate** | **p-value** | **Adj R^2^** | **Model** **p-value** |
| --- | --- | --- | --- | --- | --- |
| Log10  (volume/ quadrat +1) | Category*  Season | 0.12 | < 0.0001 | 0.13 | < 0.0001 |
|  | Season | -0.05 | 0.09 |  |  |
|  | Category | -0.35 | 0.92 |  |  |
|  | Total Quadrats | 0.01 | 0.22 |  |  |
|  | **Random Effect** | **Variance Ratio** | **DF** |  |  |
|  | Site | 0.07 | 14 |  |  |

**Table S16** Generalized linear modeling showing the relationship between site (a) total sugar (g) and (b) total nectar volume (mL) with site category (occupied, formerly occupied, previous release, and restoration), season (spring and summer), and the interaction between site category and season. Parameter estimates were averaged (mean) across all effects. Statistical significance set at the α = 0.05 level.

1. Total sugar availability

| **Dependent Variable** | **Fixed Effect** | **Estimate** | **p-value** | **Adj R^2^** | **Model** **p-value** |
| --- | --- | --- | --- | --- | --- |
| Log10 (Total Sugar) | Category*Season | 0.59 | 0.002 | 0.33 | 0.005 |
|  | Category | -0.06 | 0.04 |  |  |
|  | Season | -0.14 | 0.24 |  |  |

1. Total nectar volume

| **Dependent Variable** | **Fixed Effect** | **Estimate** | **p-value** | **Adj R^2^** | **Model** **p-value** |
| --- | --- | --- | --- | --- | --- |
| Log10 (Total Volume) | Category*Season | 0.40 | 0.002 | 0.33 | 0.005 |
|  | Category | -0.06 | 0.05 |  |  |
|  | Season | -0.09 | 0.30 |  |  |

**Table S17.** Tukey-Kramer HSD all pairs test to compare the number of flowering stems and nectar sugar (mg) per 0.5 m^2^ quadrat between categories within the spring and summer vegetation survey. Analysis of sample populations with flowering stems characterized for floral and sugar abundance. Empty quadrats and species not characterized for resource availability were not included. Data received a Log10 transformation.

**Notes S1** Sample comparisons for *Rubus flagellaris* and *Coreopsis lanceolata* samples from the field and greenhouse

When we compared samples collected from field sites and the greenhouse to assess potential maternal environmental effects on nectar composition, we found *Rubus flagellaris* samples from the field and greenhouse had similar nectar volumes (Table 9 in Day (2020), Wilcoxon Kruskal-Wallis X2 = 1.59, DF = 1, p = 0.21). However, sugar concentration was significantly different between *R. flagellaris* collection sources (X2 = 8.80, DF = 1, p = 0.003). A greater nectar sugar concentration is consistent with a lack of canopy cover and increased temperature/humidity within the greenhouse setting. Field locations with 0% canopy ranged between 6 – 50% Brix while greenhouse samples ranged between 23 – 96% Brix. Samples collected for *R. flagellaris* (n = 52) had no outliers present within the data; therefore, greenhouse samples were included in the species nectar analyses.

*Coreopsis lanceolata* samples collected from the field (n = 5) and greenhouse (n = 9) did not significantly differences in volume or sugar concentration (X2 = 1.00, DF = 1, p = 0.32; X2 = 3.39, DF = 1, p = 0.06). *C. lanceolata* was one of seven species in this study previously characterized for nectar quality by Arnold and Michaels (2017). All samples collected for this study of *C. lanceolata* (n = 14) were compared to those from that study (n = 5) to ensure consistent collection methods and compare potential maternal environmental effects (Table 10, in Day (2020). No significant difference was found between study samples in volume nor sugar concentration (X2 = 0.27, DF = 1, p = 0.60; X2 = 0.002, DF = 1, p = 0.96) supporting the inclusion of new nectar data with those previously reported.
